# Differential binding of neutralizing and non-neutralizing antibodies to native-like soluble HIV-1 Env trimers, uncleaved Env proteins, and monomeric subunits

**DOI:** 10.1186/1742-4690-11-41

**Published:** 2014-05-29

**Authors:** Anila Yasmeen, Rajesh Ringe, Ronald Derking, Albert Cupo, Jean-Philippe Julien, Dennis R Burton, Andrew B Ward, Ian A Wilson, Rogier W Sanders, John P Moore, Per Johan Klasse

**Affiliations:** 1 Department of Microbiology and Immunology, Weill Cornell Medical College, Cornell University, New York, USA; 2 Department of Medical Microbiology, Academic Medical Center, Amsterdam, the Netherlands; 3 Department of Integrative Structural and Computational Biology, International AIDS Vaccine Initiative Neutralizing Antibody Center and Center for HIV/AIDS Vaccine Immunology & Immunogen Discovery, The Scripps Research Institute, La Jolla, USA; 4 Department of Immunology and Microbial Science, International AIDS Vaccine Initiative Neutralizing Antibody Center, The Scripps Research Institute, La Jolla, CA, USA; 5 The Ragon Institute of Massachusetts General Hospital, Massachusetts Institute of Technology and Harvard University, Boston, MA, USA; 6 Skaggs Institute for Chemical Biology, The Scripps Research Institute, La Jolla, CA, USA

## Abstract

**Background:**

The trimeric envelope glycoproteins (Env) on the surface of HIV-1 virions are the targets for neutralizing antibodies (NAbs). No candidate HIV-1 immunogen has yet induced potent, broadly active NAbs (bNAbs). Part of the explanation may be that previously tested Env proteins inadequately mimic the functional, native Env complex. Trimerization and the proteolytic processing of Env precursors into gp120 and gp41 profoundly alter antigenicity, but soluble cleaved trimers are too unstable to serve as immunogens. By introducing stabilizing mutations (SOSIP), we constructed soluble, cleaved Env trimers derived from the HIV-1 subtype A isolate BG505 that resemble native Env spikes on virions both structurally and antigenically.

**Results:**

We used surface plasmon resonance (SPR) to quantify antibody binding to different forms of BG505 Env: the proteolytically cleaved SOSIP.664 trimers, cleaved gp120-gp41_ECTO_ protomers, and gp120 monomers. Non-NAbs to the CD4-binding site bound only marginally to the trimers but equally well to gp120-gp41_ECTO_ protomers and gp120 monomers, whereas the bNAb VRC01, directed to the CD4bs, bound to all three forms. In contrast, bNAbs to V1V2 glycan-dependent epitopes bound preferentially (PG9 and PG16) or exclusively (PGT145) to trimers. We also explored the antigenic consequences of three different features of SOSIP.664 gp140 trimers: the engineered inter-subunit disulfide bond, the trimer-stabilizing I559P change in gp41_ECTO_, and proteolytic cleavage at the gp120-gp41_ECTO_ junction. Each of these three features incrementally promoted native-like trimer antigenicity. We compared Fab and IgG versions of bNAbs and validated a bivalent model of IgG binding. The NAbs showed widely divergent binding kinetics and degrees of binding to native-like BG505 SOSIP.664. High off-rate constants and low stoichiometric estimates of NAb binding were associated with large amounts of residual infectivity after NAb neutralization of the corresponding BG505.T332N pseudovirus.

**Conclusions:**

The antigenicity and structural integrity of cleaved BG505 SOSIP.664 trimers render these proteins good mimics of functional Env spikes on virions. In contrast, uncleaved gp140s antigenically resemble individual gp120-gp41_ECTO_ protomers and gp120 monomers, but not native trimers. Although NAb binding to functional trimers may thus be both necessary and sufficient for neutralization, the kinetics and stoichiometry of the interaction influence the neutralizing efficacy of individual NAbs.

## Background

The trimeric envelope glycoprotein (Env) spikes sparsely decorate the surface of infectious HIV-1 virions. Each trimer consists of three hetero-dimers, in which the membrane-distal subunit gp120 associates non-covalently with the transmembrane protein gp41 [1]. When the primary receptor, CD4, on the target-cell surface is ligated by Env trimers, a site for co-receptor binding is induced, allowing Env interactions with CCR5 or CXCR4. These events trigger conformational rearrangements and a refolding of Env, which drive the fusion of the viral and cellular membranes, enabling the viral core, which contains the genetic material, to enter the cytoplasm. Because the Env trimer mediates these essential functions and is exposed on the virion exterior, it is the target for neutralizing antibodies (NAbs), which prevent infection by blocking viral entry [2,3]. No HIV-1 vaccine candidate has yet induced the potent, broadly active NAbs (bNAbs) that would be required to counter circulating HIV-1 strains, which display exceptional sequence variation in the *env* gene. But all of Env is not as variable and one approach to immunogen design is to create soluble, recombinant antigenic mimics of the functional Env trimers with the goal of focusing antibody responses on conserved neutralization epitopes [4-6].

We and others have described the design, structural properties, and antigenicity of soluble Env trimers containing gp120 subunits and most of the ecto-domain of gp41 (gp41_ECTO_) [7-15] (see Methods). The most advanced version of these trimers, based on the subtype A founder virus BG505, is designated BG505 SOSIP.664 gp140 [16,17]. Three-dimensional structures at near-atomic scale resolution of this trimer in complex with Fabs of the PGT122 and PGV04 bNAbs have been obtained, respectively, by x-ray crystallography and cryo-electron microscopy (EM) [18,19].

An alternative and predominant approach to making trimeric Env proteins has been to eliminate the cleavage site between gp120 and gp41_ECTO_, yielding uncleaved gp140s (gp140_UNC_) [9,10,12,14,15,20]. Attempts have also been made to improve the properties of gp140_UNC_ proteins by adding heterologous motifs such as Foldon and T4 bacteriophage fibritin to the C- terminus of gp41_ECTO_[12,15,21]. It is now clear, however, that the purified fraction with the mass of a trimer from various uncleaved gp140s contains predominantly aberrant, non-native structures in which three gp120 subunits dangle off a central, post-fusion 6-helix bundle formed by the gp41 part of the gp140_UNC_ protein [22,23].

Our goal is to design Env-based immunogens that most closely mimic the native form of Env found on the virion surface, so as to enhance the possibilities of inducing strong bNAb responses. Such responses emerge in only a minority of subjects after several years of HIV-1 infection [24]. In contrast, many anti-Env antibodies that arise during infection are non-neutralizing and recognize only non-native forms of Env, probably because they are elicited by shed gp120 and other non-functional or degraded Env proteins [25]. Even infectious virions harbor a mixture of functional trimers and non-native forms of Env, some trimeric, others not [26,27]. Among primary HIV-1 isolates, neutralization correlates poorly with antibody binding to monomeric gp120 but agrees well with binding to native trimers [28,29]. The explanation is that many epitopes on the trimers of primary isolates are shielded by Env trimerization and intra- and inter-protomer interactions involving the gp120 variable loops and glycans [18,19,30-32]. Hence, Env trimers with the highest ratio of NAb over non-NAb binding might have desirable immunogenic properties that could be further improved through knowledge of how bNAbs emerge during infection.

The BG505 SOSIP.664 gp140 trimers are good antigenic mimics of native Env spikes in that they occlude most non-NAb epitopes but display trimer-dependent and other bNAb epitopes well [17]. Those antigenic properties are contingent upon cleavage between the gp120 and gp41_ECTO_ subunits [23,33-35]. Here, we characterize the antigenicity of BG505 SOSIP.664 trimers by surface plasmon resonance (SPR), showing that different bNAbs bind with widely divergent kinetics. We also compare bNAb and non-NAb binding to the trimer, the previously never studied gp120-gp41_ECTO_ protomer (see Additional file 1: Figure S1), and the gp120 monomer; we dissect how the cleavage-enhancing and trimer-stabilizing features affect the antigenicity of the trimers; and we explore binding differences between monovalent Fabs and bivalent IgG. We compare these findings with the thermodynamics of bNAb binding analyzed by isothermal calorimetry (ITC) and with the high-resolution EM and x-ray crystallographic structures of Fab-trimer complexes [18,19]. We found a good agreement between the stoichiometries of Fab binding per trimer determined by other methods and the estimates derived from SPR data. We suggest that stoichiometry together with the off-rate constant of NAb binding influences the efficacy of neutralization.

## Results and discussion

### Effects of oligomerization on the antigenicity of Env trimers

**Figure 1:**
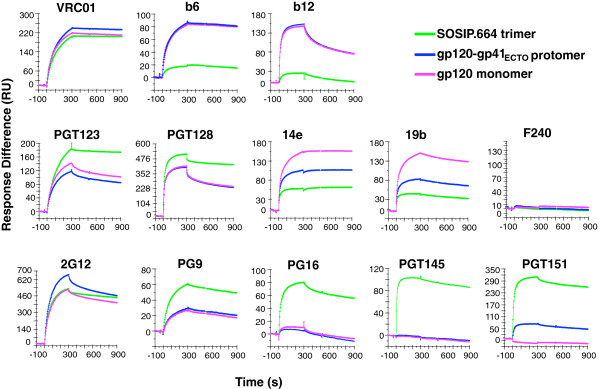
**The effect of oligomerization on Env antigenicity.** The sensorgrams show the binding (RU) of the listed IgGs to the BG505 SOSIP.664 trimer, gp120-gp41_ECTO_ protomer (gp140), and monomeric gp120 over time (s) on the x axis. Association was followed for 5 min and dissociation for 10 min. The Env proteins were captured on the chip by amine-coupled D7324 antibody. For each Ab tested similar levels of Env were captured: *R*_*L*_ values were ~ 500 RU for trimers and protomers for all Abs except for the V3-specific ones, where *R*_*L*_ was ~200 RU for trimer and protomer, and in all cases ~15% lower for gp120 to yield approximately equal numbers of gp120 subunits for all three forms of Env. The antibodies tested as analytes bind to different clusters of epitopes: b12, b6, and VRC01 to the CD4bs; F240 to cluster I in gp41; PG9, PG16, and PGT145 to V1V2-glycan epitopes at the apex of the trimer; PGT151 to a newly discovered epitope that spans the interface between gp120 and gp41_ECTO_ in one protomer and also makes contact with a second gp41_ECTO_ subunit; 2G12 to a mannose-glycan-dependent epitope; PGT123 and PGT128 to composite V3-base and glycan epitopes; and 14e and 19b to V3 epitopes. MAbs b12, b6, F240, 14e, and 19b do not neutralize the corresponding BG505.T332N virus, whereas VRC01, 2G12, PGT123, PGT128, PG9, PG16, PGT145, and PGT151 do. All MAbs were injected at 1 μM. The sensorgrams show one of two replicates.

We compared antibody binding to the BG505 SOSIP.664 trimer, the corresponding disulfide-stabilized gp120-gp41_ECTO_ protomer, which has previously never been included in NAb binding studies, and the gp120 monomer (see Methods and Additional file 1: Figure S1 for a description of the protomer). All three antigens were captured onto SPR chips at levels corresponding to approximately the same amount of immobilized gp120 (see Methods for different approaches to immobilization and the reasons for using them and Additional file 2: Table S1 for analyses of the reproducibility of capture levels). The CD4bs-directed bNAb VRC01, which potently neutralizes the corresponding BG505.T332N pseudovirus, bound strongly and similarly to all three antigens, with markedly slow dissociation. In contrast, the non-NAb b6 and the bNAb b12, also directed to the CD4bs but which do not neutralize the BG505.T332N pseudovirus [17], bound well and indistinguishably to the gp120-gp41_ECTO_ protomer and gp120 monomer, but negligibly to the trimer (Figure 1). Thus, the lack of neutralization is due to the trimerization-dependent shielding of epitopes on the gp120 subunits. The binding kinetics, however, differed between these two antibodies in that b12 dissociated markedly faster. The non-NAb F240 reacted with neither trimer nor protomer, which was as expected since its gp41_ECTO_ epitope is located in cluster I, a region interacting with gp120. Monomeric gp120 served as a negative control for F240 binding. (That the requisite gp41 sequence is present is shown later with uncleaved Env constructs).

The 2G12 bNAb bound similarly to its glycan-dependent outer-domain epitope on the gp120 monomer and trimer, and marginally better to the protomer. The two glycan- and V3-base-dependent bNAbs PGT123 and PGT128 recognized the trimer somewhat better than protomer and gp120, with faster dissociation from the latter two.

The two V3-specific MAbs 14e and 19b, which do not neutralize BG505.T332N pseudovirus [17], bound strongly to the gp120 monomer but only negligibly to the trimer. Plausible mechanisms of the shielding of V3 epitopes include burial of V3 in the trimer interface and masking by V1V2 [18,19]. Unexpectedly, both MAbs reacted with the gp120-gp41_ECTO_ protomer substantially less well than with the gp120 monomer (although better than with the trimer). The gp41_ECTO_ moiety would therefore seem to contribute, through intra-protomeric conformational effects, to the nearly complete shielding of these V3 epitopes on the SOSIP.664 trimer immobilized on the SPR chip. There are precedents for antigenic effects of gp41 on gp120 epitopes. For example, a neutralization-escape mutation in cluster I reduces the sensitivity of the T-cell line-adapted virus HxB2 to CD4bs-directed Abs [36-38]; conversely, cluster-I mutations in primary isolates can confer or contribute to sensitivity to neutralization by sCD4, b12, or plasma from HIV-1-infected people [39,40]. Furthermore, substitutions in the MPER can strongly affect viral sensitivity to V3 NAbs [41,42].

To investigate the effect of gp41_ECTO_ on V3 antigenicity, we used untagged gp120-gp41_ECTO_ protomer and gp120 monomer as analytes and compared their binding to anti-Fc-immobilized MAbs 14e and 19b. In that format, the anti-V3 MAbs did not distinguish quite as clearly between the gp120 monomer and gp120-gp41_ECTO_ protomer (Additional file 1: Figure S2). Therefore, one explanation for the lower binding of the V3 MAbs to the gp120-gp41_ECTO_ protomer than to gp120 monomers, under the conditions of Figure 1, might be that immobilized protomer molecules interact with each other and shield the 14e and 19b epitopes [18,19]. The D7324 epitope-tag or the immobilization might also indirectly affect the exposure or conformation of V3. We also note that the extent of 14e and 19b binding to the BG505 SOSIP.664 trimer is assay-dependent: the V3 region is more accessible in a D7324-capture ELISA than in SPR or electron microscopy [17]. Overall, there is still much to be learned about what determines the degree of V3 exposure on different forms of Env and how those factors affect immunogenicity.

We confirmed that the bNAbs PG9, PG16, and PGT145, directed to V1V2 quaternary-dependent epitopes, bind better to the trimer than to the gp120 monomer [16,43,44]. PG16 dissociated faster from the trimer than PG9 and PGT145 (Figure 1). While PG9 and PG16 did react to an extent with gp120 and the gp120-gp41_ECTO_ protomer, PGT145 bound to neither of those, only to the trimer. PG9 is known to bind to some gp120 monomers, particularly to BG505 gp120 [45]. Here, PG9 bound indistinguishably to gp120 and the gp120-gp41_ECTO_ protomer, although much more weakly than to the trimer. Hence, the presence of gp41_ECTO_ enhances reactivity with PG9, PG16, and PGT145, not by affecting the conformation of individual gp120 subunits independently of the trimeric context, but by mediating trimerization and thus creating the complete epitope described for this group of bNAbs [16].

The recently described bNAb PGT151, directed to a novel epitope at the gp120-gp41 interface [46,47], had a different binding profile. It failed to bind gp120 but did react with the gp120-gp41_ECTO_ protomer, although less well than with the trimer (Figure 1). This reactivity profile is consistent with the demarcation of the PGT151 epitope, which involves both gp120 and gp41_ECTO_ components of one protomer and also a second gp41_ECTO_ subunit [46,47].

In summary, whereas trimerization shields non-NAb epitopes, it is necessary for creating, or optimizing, the epitopes for several bNAbs. Thus, all non-NAbs bound well to monomeric forms of Env but not to the trimer, while the trimer-specific bNAbs exhibited the inverse pattern. The gp41_ECTO_ moiety within each protomer did not influence the antigenicity of most gp120 epitopes, including the CD4bs, but it reduced binding somewhat to the V3 region on the gp120-gp41_ECTO_ protomers.

### Effects of proteolytic cleavage and stabilizing mutations on antibody binding to Env trimers

Recently, we showed that cleavage at the junction between gp120 and gp41_ECTO_ strongly promotes a native-like structure of the BG505 SOSIP.664 gp140 trimer, as determined by negative-stain EM, and that the compact, native-like trimer binds NAbs but not non-NAbs [23]. Likewise, other biophysical techniques reveal the non-native structure of uncleaved oligomeric gp140s [22]. The preferential binding of NAbs to proteolytically processed Env on the cell surface is also well-described [34]. Here, we extend those observations by further dissecting what modifications of BG505 SOSIP.664 Env are responsible for which antigenic effects.

**Figure 2:**
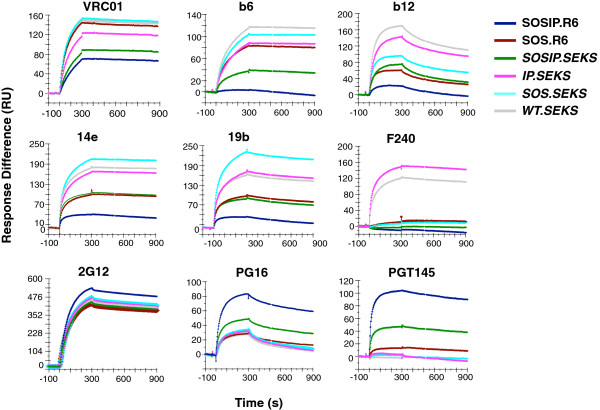
**The effects of cleavage and trimer-stabilizing mutations on Env antigenicity.** The sensorgrams show the binding (RU) of the listed MAbs to BG505 SOSIP.664 trimers (labeled “SOSIP.R6”) and five mutated variants thereof (see Results). MAbs b12, b6, F240, 14e, and 19b do not neutralize the corresponding BG505.T332N virus, whereas VRC01, 2G12, PG16, and PGT145 all do. The SPR method was the same as for Figure 1, except that the MAbs were injected at 500 nM. The sensorgrams show one of two replicates.

We studied the binding of nine MAbs to six forms of Env: SOSIP.R6 (i.e., SOSIP.664) is the fully cleaved and stabilized form; *WT.SEKS* lacks both the SOSIP mutations and the cleavage site; *SOSIP.SEKS* lacks only the cleavage site; SOS.R6 lacks only the I559P mutation; *SOS.SEKS* lacks the I559P mutation and the cleavage site; *IP.SEKS* lacks the SOS link between gp120 and gp41_ECTO_ as well as the cleavage site (Figure 2). Note that cleaved Env lacking the SOS linkage could not be studied because with that construct gp120 completely dissociates from gp41_ECTO_.

VRC01, a CD4bs bNAb that does neutralize the BG505.T332N pseudovirus, bound significantly to all six forms of Env (Figure 2), while clearly differentiating among them. The weakest binding, while still substantial, occurred with the most stabilized, native-like form of Env, SOSIP.R6. Eliminating only the cleavage site (*SOSIP.SEKS*) marginally increased binding, whereas reverting the SOS mutations and removing the cleavage site (*IP.SEKS*) increased binding further. The three highest binding curves were recorded for SOS.R6, *WT.SEKS*, and *SOS.SEKS*, with small increments in that order. Overall, the more compact trimer structure that is contingent on cleavage is compatible with binding, while posing a limited impediment.

Three MAbs (the CD4bs-specific b6, b12, and the gp41-directed F240) that do not neutralize BG505 had distinct sensitivities to Env modifications. All three MAbs strongly bound to *WT.SEKS*, although b12 dissociated faster than b6 and F240; none of them bound to SOSIP.R6, the trimer with the most native-like structure; and b6 and b12 bound at intermediate levels to *SOSIP.SEKS*. The latter result shows that, even in the absence of cleavage, the trimer-stabilizing modifications partially mask these overlapping epitopes. The I559P mutation had a weak but definite masking effect on both the b6 and b12 epitopes when Env was uncleaved (*IP.SEKS vs. WT.SEKS*). The SOS modification had a stronger masking effect for b12 than b6 on uncleaved Env (*SOS.SEKS vs. WT.SEKS*). Furthermore, in the cleaved SOS context, absence of the I559P modification had differential effects on b6 and b12 binding: b6 bound to SOS.R6 more strongly than b12 (SOS.R6 *vs.* SOSIP.R6). Hence, the I559P point substitution in gp41_ECTO_ influences the CD4bs. It can be noted that although neither b6 nor b12 neutralizes the BG505.T332N pseudovirus, they have drastically different properties and modes of Env interaction in that b6 is a non-NAb and b12 a bNAb. It is therefore significant that these two epitopes, overlapping the CD4bs, are differentially affected by how gp120 is anchored to gp41_ECTO_, and also by the presence of the trimer-stabilizing I559P change.

None of the Env variants containing the SOS modification bound the F240 non-NAb against an epitope in cluster I of gp41_ECTO_. Either gp120, when disulfide-linked to gp41_ECTO_, masks the F240 epitope, or the disulfide bond to C605 (or just the Cys side chain itself), disrupts the epitope. The I559P change in the gp140_UNC_ context enhanced F240 binding (*IP.SEKS* compared with *WT.SEKS*, which differ only at residue-559, Figure 2). The I559P change probably impedes the formation of a six-helix bundle and thereby favors F240 binding.

The binding of 2G12 to the different forms of Env was similar, which shows that its gp120 epitope is affected by neither cleavage nor the SOSIP modifications (since all Env variants were captured to near-identical levels as shown by the *R*_*L*_ values in Additional file 2: Table S1). The two V3-directed non-NAbs 14e and 19b bound only weakly (<50 RU) to the native-like SOSIP.R6 trimers. They both bound to intermediate levels with SOS.R6 and *SOSIP.SEKS*, indicating that the omission of the I559P change and the lack of cleavage equally increased exposure of the V3 region. The two V3 MAbs bound to high and similar levels to *WT.SEKS* and *IP.SEKS*, but yielded even higher levels with *SOS.SEKS*. When added to *WT.SEKS*, I559P alone had no effect, but added to *SOS.SEKS* it reduced binding markedly. In summary, the I559P change in the cleaved context and cleavage itself both markedly shield V3 epitopes, whereas in the uncleaved context SOS must be combined with the I559P change to exert a V3 masking effect. Note that the reduced binding of these MAbs to the gp120-gp41_ECTO_ protomer, compared with gp120, also indicated that gp41_ECTO_ exerts indirect effects on V3 antigenicity (Figure 1). Overall, however, the Env form that least exposes V3 non-NAb epitopes is the cleaved, native-like trimer SOSIP.R6 (elsewhere referred to as SOSIP.664).

Two quaternary structure-dependent bNAbs, PG16 and PGT145, were also studied. PG16 bound strongly only to SOSIP.R6. It did not recognize the *WT.SEKS* uncleaved gp140, but bound to intermediate levels when the SOSIP modifications were present in the uncleaved gp140, *SOSIP.SEKS*. Both *IP.SEKS* and SOS.R6, which share no modifications, bound PG16 to low levels. The I559P change therefore strongly promotes PG16 binding only when Env is cleaved. For PGT145, the differences between the strong, high-level binding to the native-like SOSIP.R6 trimers and the other Env variants were even starker than for PG16. Thus, PGT145 bound partly to *SOSIP.SEKS*, but negligibly to the other variants. In summary, for full PGT145 binding, cleavage, SOS, and 1559P are all necessary. The PGT145 reactivities with the different Env variants are strikingly congruent with the estimated proportions of native-like trimers present, as determined by negative stain-EM. Thus, apart from SOSIP.R6, which yields close to 100% native-like trimers, only the *SOSIP.SEKS* construct yields more than a few percent of native-like trimers [23]. Hence the antigenicity of *SOSIP.SEKS* reflects its mixed population of native-like trimers and structurally aberrant forms of Env [23].

### Kinetic modeling of and stoichimetric estimates of monovalent and bivalent binding to Env

There are advantages to studying both monovalent Fabs and bivalent IgG. The latter is the natural antibody form, and therefore of greater relevance to blocking viral entry *in vivo*. But the degree of bivalent binding to Env trimers on the virion is uncertain, and it has been argued that HIV incorporates exceedingly few Env spikes, thereby disfavoring bivalent NAb binding [48]. Such an escape strategy could, however, be a double-edged sword. On the one hand, by having few trimers on its surface, and hence long average distances between them, HIV-1 would minimize the enhancement of neutralization potency that NAb avidity confers [48,49]. On the other hand, an opposing effect would arise, namely that the fewer spare trimers virions have, above what is necessary for infection, the lower would be the minimal occupancy required for neutralization, an effect making the virus more vulnerable [50-54].

The spacing and orientation of the epitopes studied here preclude the bridging of two epitopes on the same trimer by one IgG molecule, i.e. intra-trimeric bivalent binding; and for the NAbs PG9, PG16, and PGT145, directed to the trimer apex, the unusual stoichiometry of one paratope per trimer already excludes their intra-trimeric bivalent binding [16,18]. Whether an IgG of any specificity can bridge two epitopes on the same trimer is doubtful [48]. The PGT122 Fab binds with an angle that is incompatible with intra-trimeric cross-linking of epitopes by the corresponding IgG. Still, its angle of binding is deemed less unfavorable for intra-trimeric bivalent binding than that of any other Fabs studied [18]. Thus, even for NAbs that, unlike PG9, PG16, and PGT145, can potentially occupy three epitopes per trimer, only inter-trimeric bivalent binding needs to be considered.

We do not know how many functional or defective trimers the average infectious virion or pseudovirion carries. That number is likely to vary over the virion population and the proportion of functional trimers will decline as the virions decay. A cryo-EM study of the T-cell line-adapted isolate MN, however, observed a range of 4 to 35 Env spikes per virion, with an average of 14 [55]. The range may stretch higher for primary isolates, and virions with the fewest spikes may not be infectious. Furthermore, the most frequent nearest-neighbor spacing of spikes was ~ 15 nm, and the spikes were not randomly distributed over the virion surface but tended to cluster. Therefore, the maximum distance from paratope to paratope of an IgG molecule would sometimes suffice for spanning adjacent spikes. For comparison, the density of Env we used on the chips in standard experiments was ~ 700 trimer molecules per μm^2^, which corresponds to a density between the average of 14 and the top value of 35 per virion (see SI for calculations). Hence, although the different kinds of Env immobilization for SPR used here differ qualitatively from each other from how trimers decorate the virion surface (see SI), they are quantitatively relevant to neutralization. We therefore considered it worthwhile to explore mixed bivalent-monovalent binding models by SPR, and assess how closely the monovalent binding by IgG resembles the binding by Fabs.

We found that the bivalent model identifies a genuine strengthening of binding by the IgGs due to two-point binding. Five lines of evidence indicate this: Fab and IgG comparisons, the relative goodness of fits by Langmuir and bivalent models, the relative significances of the kinetic parameter values, the influence of ligand density, and the analyte-concentration dependence of the size of the bivalent component (Additional file 3: Tables S2 and S3, Additional file 1: Figures S3, S4, S5). Unfortunately, no comparison with other methods for further validation of bivalent binding to similarly immobilized Env proteins is readily available. We can nevertheless conclude that both monovalent and bivalent binding can be measured by SPR, although some questions remain about how accurately the bivalent model attributes the avidity effect to the second binding event.

The *S*_*m*_ value obtained by SPR is a stoichiometric estimate that primarily serves the purpose of quality control of the ligand; if only a small fraction of immobilized trimers were able to bind NAbs, it would indicate that the trimers were structurally compromised before or after immobilization. We obtained reassuringly high stoichiometric estimates (Additional file 3: Table S4), indicating that the majorities of the Env molecules (trimers and protomers) were structurally intact. In addition we found generally good agreement between the *S*_*m*_ values and the stoichiometries observed by ITC and EM. For several reasons (see the SI), however, the accuracy and precision of *S*_*m*_ values can be questioned. We therefore compared three methods of assessing the stoichiometry and found that the measurements were quite robust (Additional file 3: Table S4). Furthermore, the convergence of *S*_*m*_ estimates for Fabs and IgGs directed to the same epitope validates the bivalent modeling. We conclude that SPR can measure bivalent binding and yield reasonable *S*_*m*_ estimates, which complement the stoichiometric measurements obtained by other methods.

### Kinetic differentiation of NAb binding to SOSIP.664 trimers and gp120-gp41_ECTO_ protomers

**Figure 3:**
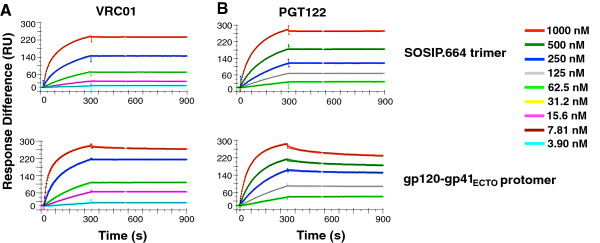
**Comparisons of VRC01 and PGT122 binding to the Env SOSIP.664 trimer and gp120-gp41**_**ECTO **_**protomer.** The sensorgrams show VRC01 **(A)** and PGT122 **(B)** IgG binding to BG505 SOSIP.664 trimers (top) and gp120-gp41_ECTO_ protomers (bottom). The colored curves show the response at various analyte concentrations as indicated to the right. Note that the color code is the same for all diagrams but that the titration ranges start and end at different concentrations and also differ in the dilution steps. The modeled curves in black (bivalent model) become visible only when they diverge from the empirical data. The sensorgrams show one of two of replicates. In some experiments the dissociation phase had to be extended to 20 min to achieve significant values (T > 10) for *k*_*d1*_ (not shown).

The kinetic profiles of VRC01 and PGT122, two NAbs that bound well to both SOSIP.664 trimers and gp120-gp41_ECTO_ protomers, are displayed in Figure 3. (As shown in Figure 1, VRC01 only marginally differentiated among trimer, protomer, and gp120 in single-concentration qualitative analysis, whereas two NAbs, PGT123 and PGT128, closely related to PGT122, showed a slight trimer preference). The comparison by full kinetic modeling revealed differences in how VRC01 and PGT122 bind to the two forms of Env. VRC01 bound to the SOSIP.664 trimers with moderately fast association and markedly slow dissociation; but as it both associated faster with and dissociated more slowly from the protomer, its affinity was 10-fold higher for the protomer than the trimer (Additional file 3: Table S5). This affinity difference between trimer and protomer is in line with the results in Figure 2, which suggests that the formation of stable, native-like trimers disfavors VRC01 binding. The affinity difference is explained mechanistically by the three-dimensional cryo-EM structure of the same trimer in complex with the Fab of the CD4bs NAb PGV04. Thus, one protomer restricts access of the Fab to the CD4bs on the neighboring protomer [19].

PGT122 showed the opposite preference. Although its on-rate constants for binding to trimer and protomer were similar, the off-rate constant was markedly lower for the trimer than protomer. Therefore the affinity of the intial interaction (before bivalent strengthening) was significantly higher for the trimer than the protomer (Additional file 3: Table S5). This affinity difference might be explained by the three-dimensional crystallographic structure of the BG505 SOSIP.664 trimer in complex with the PGT122 Fab, which shows the intricate relationship of the PGT122 epitope with the apex of the trimer where the protomers interact through the V1V2 and V3 variable regions [18]. Furthermore, it can be noted that intermediates between germline and mature versions of the bNAbs PGT121-134 bind better to cell-surface expressed Env than to monomeric gp120, another indication of a certain degree of trimer preference for these NAbs [56].

### The binding of NAbs to the SOSIP.664 trimer

**Figure 4:**
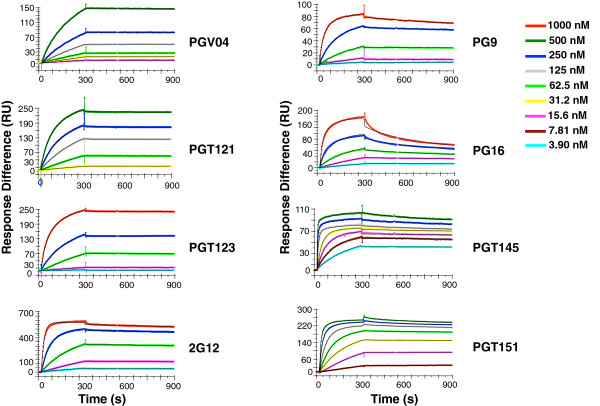
**The kinetics of bNAb interactions with SOSIP.664 trimers.** The sensorgrams show binding titrations fitted with the bivalent model for all NAbs except 2G12 (Langmuir model). The colored curves show the response at analyte concentrations indicated to the right. Note that the color code is the same for all diagrams but that the titration ranges start and end at different concentrations and also differ in the dilution steps. The modeled curves in black are only visible when they diverge from the data. The sensorgrams show one of the multiples of replicates (n) given in Additional file 3: Table S5. In some experiments the dissociation phase had to be extended to 20 min to achieve significant values (T > 10) for *k*_*d1*_ (not shown).

Binding profiles of IgG versions of NAbs against the trimer are shown in Figure 4 with the kinetic-modeling results in Additional file 3: Table S5. A subset of the NAbs were also studied as Fabs (Additional file 1: Figure S5). The stoichometric estimates for IgGs and Fabs are given in Additional file 3: Table S4. Notably, the stoichiometric estimates for the IgGs obtained by the bivalent modeling are given as the number of paratopes bound per Env molecule (trimer or protomer). Hence the *S*_*m*_ values for IgG and Fab are directly comparable (see Additional file 4: Supplementary results and commentary).

The stoichiometric *S*_*m*_ estimates by SPR for PGV04 IgG and Fab, 1.5 and 1.8, respectively (Additional file 3: Table S4), fall between those obtained by ITC (1.3) and EM (average 2.2) with the same trimers. In the EM analysis of bound PGV04 Fabs, ~44% of the trimer molecules were occupied by three Fabs, whereas smaller sub-populations had two, one, or no Fab bound [19]. Causes of the binding restrictions might be differential glycosylation and glycan processing. Indeed, when the trimers were deglycosylated by Endo H, the stoichiometry of PGV04 Fab binding determined by ITC increased from 1.3 to 2.0 [19].

The *S*_*m*_ estimates for PGT121-3 showed different degrees of variation among the three methods of deriving them from the SPR data (1.6-2.8, 1.6-2.5, and 1.9-2.0, respectively; Additional file 3: Table S4). For comparison, crystallography has demonstrated that three Fabs of PGT122, which is similar to PGT121 and PGT123, can bind to the BG505 SOSIP.664 trimer, whereas for PGT121 the stoichiometry determined by ITC was 2.4 Fabs per trimer [17,18]. Again, variations in glycan composition might explain binding restrictions.

Unlike the other IgGs studied here, 2G12 lacks the capacity for functionally bivalent binding because of an unusual domain-swap structure that unites the two inflexible Fab arms into one binding site [57]. Accordingly, its binding was fitted with the Langmuir model. The *S*_*m*_ value was 2.9 for the 2G12-trimer interaction (Additional file 3: Table S4), indicative of nearly complete occupancy and somewhat higher than the value obtained by ITC, 2.4 [17].

The NAbs PG9, PG16, and PGT145, directed to broadly similar, quaternary structure-dependent V1V2 glycan epitopes, have the unusual maximum stoichiometry of a single Fab per trimer [16]. By SPR, the stoichiometric estimate was close to 1 for all three IgG versions (0.97 for PG9; 0.96 for PG16; and 0.78 for PGT145 by the most precise method) and somewhat lower for PGT145 Fab, 0.60 (Additional file 3: Table S4). For comparison, the stoichiometry of PG9 Fab obtained by ITC was 0.8 [17]. Notably, SPR suggested that PG9, PG16, and PGT145 bound to trimers with stoichiometries similar to those of VRC01 and PGT122 to protomers, which agrees with established stoichiometries and thereby validates the estimates [16,18,19,58,59].

PGT151 bound with an estimated stoichiometry of 2 paratopes per trimer (Additional file 3: Table S4), in agreement with the recent EM data on how this new bNAb recognizes an epitope formed by contributions from one gp120 monomer and two gp41 subunits [46,47]; the ITC-derived stoichiometric value, 1.3, was somewhat lower [46].

The kinetic profiles differed widely among the NAbs, also among those directed to overlapping epitopes (Figure 4 and Additional file 3: Table S5). The on-rate constant of the monovalent component for the binding to trimer, *k*_*on1*_, varied 62-fold; the off-rate constant, *k*_*off1*_, varied more, 570-fold, whereas the ratio of these two parameters, i.e., the dissociation constant, *K*_*d1*_, varied 380-fold. That the combined variation was relatively limited reflects a positive, albeit weak, correlation of the two kinetic constants (r = 0.67, p = 0.05). Since the two constants would diverge during affinity maturation, their correlation suggests immunological and chemical impediments that should ideally be overcome when designing vaccination strategies. We return to the apparent influence of the most variable parameter, *k*_*off1*_*,* below.

A direct comparison of potency in monovalent binding and virus neutralization was only possible for the small-subset of NAbs that we studied also as Fabs, but some findings are noteworthy. The SPR-derived *K*_*d*_ value for PGV04 Fab (7.7 nM, Additional file 3: Table S3) was lower than the corresponding *K*_*d*_ value obtained by ITC, 155 nM [19]. Likewise, the *K*_*d1*_ value for PGT121 IgG (0.76 nM, Additional file 3: Table S5) was considerably lower than the ITC-derived value of *K*_*d*_ for its Fab (151 nM [17]). It is uncertain whether these discrepancies for PGV04 and PGT121 reflect genuine differences in affinity between binding to tagged immobilized and untagged solution-phase trimers, or between the intrinsic affinity of IgGs and Fabs for the trimer ([60], see further discussion in Additional file 4: Supplementary results and commentary). It should be noted, however, that the IC_50_ values for neutralization of the BG505.T332N pseudovirus by the PGV04 and PGT121 Fabs were 3.5 and 1.1 nM, i.e. close to the SPR *K*_*d*_ and *K*_*d1*_ values (Additional file 3: Table S6). If Fabs have the same affinity for SOSIP.664 trimers as for functional spikes on virions, the *K*_*d*_ values obtained SPR would agree with observations and modeling of neutralizing occupancies [2,29,50-53]. The ITC values suggest substantial affinity differences between the soluble and the virion-anchored native trimers, or else neutralization potency should be considerably lower. Hence, it will be important to understand the bases for these measurements to allow optimal mimicry of native trimers.

The *K*_*d*_ for the PGT123 Fab derived by SPR (5.1 nM, Additional file 3: Table S3) and the corresponding IC_50_ value (2.1 nM) were also close (Additional file 3: Tables S3 and S6). 2G12 had a somewhat lower *K*_*d*_ by SPR, 1.3 nM, than its IC_50_ value (5.1 nM), but as has been noted, the affinity for the 2G12-affinity-purified trimer is expected to be higher than for the average native trimer on virions [17]. For comparison, the *K*_*d*_ for 2G12 obtained by ITC was 16 nM, although a lower-affinity interaction was also detected with a *K*_*d*_ *~* 12 μM.

The IC_50_ for PGT145 Fab neutralization of BG505.T332N pseudovirus, 2.7 nM, was close to the *K*_*d*_ for the Fab, 2.0 nM (and to *K*_*d1*_ for monovalent binding of IgG, 2.9 nM); if the measured affinity were relevant, 50% neutralization and 50% occupancy of Fab on trimer would approximately coincide (Additional file 3: Tables S3 and S6). This agrees with modeling of neutralization data for other HIV-1 strains [50,52].

A greater discrepancy between binding and neutralization was observed for PGT151 than for the other NAbs. The *K*_*d1*_ for the monovalent component of IgG binding to trimer was 6.3 nM; the *K*_*d*_ for Fab was 7.2 nM, i.e. the two dissociation constants agreed excellently. In contrast, the IC_50_ for IgG was markedly low, 0.010 nM (no Fab neutralization data were available), lower than would be explained by the avidity effect, which was only ~ 10-fold for the other NAbs (Additional file 3: Table S6). Therefore it is plausible that the truncation at residue 664 and the stabilizing modifications of BG505 SOSIP.664 render the affinity of PGT151 for the soluble trimer lower than for functional spikes on the virion. Thus with the exception of PGT151, if the SPR conditions simulate binding to functional trimers, then 50% neutralization would occur in an approximate zone around 50% occupancy, which agrees with modeling of neutralization data for other HIV-1 strains [50,52].

### The persistent fraction in neutralization compared with kinetics and stoichiometry of binding to BG505 SOSIP.664 trimers

What binding properties of NAbs apart from affinity for functional Env trimers can influence how well they neutralize? Neutralization is often characterized merely in terms of potency, i.e. NAb IC_50_, but the efficacy or degree of neutralization is also important. Some NAbs, including PG16 and others to quaternary-structural epitopes, when used against particular viral isolates, yield neutralization curves with shallow slopes and low maximum plateaus [44,61]. But NAbs that give ~100% neutralization in conventional plots of relative reduction in infectivity as a function of log NAb concentration can differ widely in efficacy. Classically, the *persistent fraction* (PF) of infectivity at maximum neutralization has been measured as the logarithmic relative residual infectivity, which can range over many orders of magnitude. In contrast, conventional neutralization plots often show significant effects over less than one order of magnitude, and to quantify the PF experimentally requires a dynamic range that some neutralization assays lack. The PF has been linked to multiple properties of viruses and Abs, although Burnet originally attributed it mainly to dissociation by NAb [62-65]. Here, we focus on potential differential determinants of PF among the NAbs, rather than among properties of the virions, which were constant in these experiments. The NAbs analyzed in this manner were those for which we had obtained kinetic and stoichiometric binding data: PGV04, VRC01, 2G12, PGT121, PGT122, PGT123, PG9, PG16, PGT145, and PGT151 (Figure 4, Additional file 3: Tables S4 and S5).

**Figure 5:**
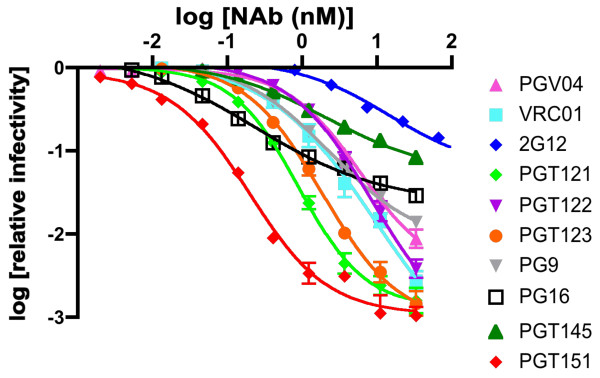
**Persistent fractions in neutralization assays.** The infectivity of BG505.T332N pseudovirus was measured on Tzm-bl cells as luciferase activity (luminescence) after incubation with NAbs. The log [relative infectivity] is expressed on the y-axis as a function of the log NAb concentration [nM] on the x-axis. The data are fitted with a sigmoid function with variable slope and an unconstrained upper plateau; the lower plateau as a fitted parameter represents the persistent fraction, PF.

We previously reported a strong correlation between NAb binding to D7324 epitope-tagged BG505 SOSIP.664 trimers in ELISA and neutralizing potency against the sequence-matched BG505.T332N pseudovirus [17]. In conventional plots they all displayed ~100% neutralization. To explore plateaus of residual infectivity we re-analyzed the same neutralization data by expressing the log of relative infectivity as a function of the log of the NAb concentration (Figure 5, Table 1). The resulting curves for most of the NAbs tended to level off, yielding widely different PFs that could be extrapolated with high precision by non-linear regression fitting of a sigmoid function to the log-log data (Table 1). We emphasize, however, that the data do not unequivocally demonstrate PFs even with the best fits. Ideally the assay should have a wider dynamic range. And an alternative to absolute plateaus is the possibility that the curves are biphasic with large slope reductions for the second part. Regardless, the data show clear deviations from what would be predicted from homogenous affinities and uniform thresholds of neutralization. For simplicity, we refer to the viral infectivity in the less effective zone of neutralization as the PF; the efficacy of neutralization is defined as the extrapolated maximum degree of inhibition of viral infectivity. Since the mannose-dependent 2G12 epitope is known to be heterogeneous, it is noteworthy that 2G12 also gave the highest PF (Figure 5, Table 1). This heterogeneity would be more pronounced among the unselected Env proteins incorporated into the pseudovirions than among the 2G12-affinity-purified trimers [17], as noted above in relation to the higher IC_50_ than *K*_*d*_ for 2G12. We therefore excluded 2G12 from further analyses.

**Table 1:** Neutralizing and binding properties of bNAbs: stoichiometry, off-rate constant, and persistent fraction (PF)

**NAb**	***S***_***m***_	***k***_***off1 ***_**(1/s)**	**Log PF**
VRC01 (n = 6)^*a*^	1.6	4.7 ^**.**^ 10^−6^	−3.7 ± 0.80
PGV04 (n = 4)	1.5	<1.0 ^**.**^ 10^−5^	−2.5 ± 0.17
PGT121 (n = 10)	1.7	8.5 ^**.**^ 10^−6^	−2.8 ± 0.089
PGT122 (n = 8)	1.6	3.0 ^**.**^ 10^−6^	−3.2 ± 0.39
PGT123 (n = 8)	2.0	9.5 ^**.**^ 10^−6^	−3.1 ± 0.14
2G12 (n = 6)	2.9	1.5 ^**.**^ 10^−4^	−1.2 ± 0.082
PG9 (n = 6)	0.97	7.1 ^**.**^ 10^−4^	−2.1 ± 0.12
PG16 (n = 8)	0.96	2.7 ^**.**^ 10^−3^	−1.6 ± 0.071
PGT145 (n = 6)	0.78	6.9 ^**.**^ 10^−4^	−1.3 ± 0.12
PGT151 (n = 6)	1.8	3.5 ^**.**^ 10^−4^	−3.0 ± 0.086

^*a*^The values in the Table are derived from global non-linear regression fits of data from n replicate neutralization titrations ± s.e.m. for the logarithmic PF values. The n values for SPR analyses are given in Additional file 3: Tables S4 and S5, where the *S*_*m*_*and k*_*off1*_ are included ± s.e.m.

We next tested the correlations of log PF with the Hill slope, EC_50_, *k*_*on1*_, *k*_*off1*_, *K*_*d1*_*, k*_*on2*_, *k*_*off2,*_*K*_*d2*_*,* and *S*_*m*_. PF correlated positively with *k*_*off1*_ (r = 0.85; p = 0.0037) and negatively with *S*_*m*_ (r = − 0.73; p = 0.025). The other parameters gave no marked or significant correlations. To test how the two parameters complemented each other, we also explored the correlation between the PF and the sum of *k*_*off1*_ ranks and inverse *S*_*m*_ ranks (which did not correlate with each other). The resulting positive correlation was even stronger and more significant (r = 0.89; two-tailed p = 0.0013). Thus, the influences of high off-rates and low stoichiometries might be reinforcing each other.

To summarize, we found that the faster the NAb dissociated after its initial encounter with the trimer, and the lower the stoichiometry (excluding 2G12), the higher was the PF. Dissociation of the NAb, in the dynamic situation of competition between NAb and receptors for binding to Env, may leave some persistent infectivity. And neutralization might be more vulnerable to dissociation when a single paratope binds to an Env trimer than when three can bind; for even when three can bind, a single bound paratope may be sufficient to inactivate the trimer [2,50,52]. Still, the relationship between stoichiometry and PF may be a mere coincidence; the real cause might be the heterogeneity of the epitopes that are affected by varied glycosylation, including in this case 2G12, which gave the highest PF.

The observed curve shapes and displacements can be explained if some degree of bivalent binding occurs with the pseudovirions; and if, close to saturation, IgG binds predominantly in a monovalent fashion in accordance with the well-established prozone effect [66]. At that high occupancy, inhibition by IgG would approach that of the Fab. Thereby, if a NAb has a strong capacity for bivalent binding because of a favorable epitope location, this will benefit potency more than efficacy.

## Conclusions

SPR has been used in multiple formats to study HIV-1 Env-NAb interactions. In some studies, Abs or Fabs have been captured and the binding of various forms of gp120 or uncleaved gp140 proteins in solution analyzed. In other studies, gp120, native-like SOSIP trimers, or uncleaved gp140s have been covalently immobilized and Fab or IgG used in solution [11,12,67-70]. Even when there is potential for more complex binding, simple Langmuir models have generally been fitted to the binding data [11,12,67-69]. These approaches have shortcomings. For example, gp120 monomers and uncleaved gp140s, which do not mimic functional spikes, have often been used; Env proteins can be distorted by direct covalent immobilization to the chips; when trimers are used in solution, weak interactions can be augmented through trivalent binding; and Fabs, although excellent tools for dissecting intrinsic affinity, bind differently from IgGs.

Here, we analyzed IgG and Fab binding to native-like BG505 SOSIP.664 trimers, which were immobilized via C-terminal tags so as to impair their antigenicity minimally, an approach we previously applied in qualitative studies [17,23]. We dissected the effects of the individual modifications that were introduced to make a stable, cleaved SOSIP trimer, compared the trimer with the corresponding gp120 monomer and gp120-gp41_ECTO_ protomer, modeled the binding kinetics and stoichiometries of a panel of bNAbs, and related these results to the persistent fraction of neutralization.

We show that proteolytic cleavage and the stabilizing modifications all contribute to the native-like antigenicity of the BG505 SOSIP.664 trimer. In many respects, uncleaved gp140, the gp120-gp41_ECTO_ protomer, and monomeric gp120 are antigenically similar, in that they bind non-NAbs strongly but interact weakly or not at all with bNAbs directed to quaternary structure-dependent epitopes at the trimer apex.

We identify a variety of kinetic profiles for the binding of different bNAbs to the native-like trimers, some featuring extremely low *k*_*off1*_ values, at or below the limit of detection (e.g., VRC01, PGV04, PGT121, PGT122, and PGT123; Figures 3 and 4; Additional file 1: Figure S5; Additional file 3: Tables S3 and S5). Of biological importance, such low off-rate constants agree with the slow genesis of NAbs through multiple rounds of somatic hypermutation in the germinal centers of lymph nodes [71-75]. When virus or Env dissociates slowly enough from B-cell receptors, the rate of internalization of the complex becomes limiting for antigen presentation to follicular T-helper cells and thereby for positive selection of increased affinity. When a new Env mutant arises, the B-cell receptor adapts through mutations, reducing *k*_*off*_ until the internalization rate again becomes limiting. Eventually the effects of such iterative selection would be reflected in the kinetics of NAb binding to heterologous Env as, for example, studied here.

The kinetic profiles for several of the NAbs we have analyzed suggest more leeway in increasing *k*_*on*_ than in lowering *k*_*off*_. Only for the PGT145 bNAb did the *k*_*on*_ verge on the diffusion limit (i.e., 10^5^-10^6^ (1/Ms)) [74,75]. One task in designing immunogens for sequential immunization is therefore to guide somatic hypermutation towards higher on-rate constants for cross-reacting NAbs, perhaps by manipulating bNAb epitope accessibility [75].

We found that fast dissociation and low stoichiometry were associated with large PFs. We argue that neutralization by bNAbs should be described not only as potency but also as efficacy, i.e. the maximum extent of inhibition. For a high PF may allow infection *in vivo*, even in the face of a potent bNAb.

Passive immunization with 2G12 or PGT121 is particularly efficient at preventing mucosal transmission in the SHIV-macaque model of HIV-1 infection [76,77]. Of course, binding properties of any bNAbs that could explain why they protect well must be shown to apply to Env from the challenge virus. Even so, the high stoichiometry and on-rate constant of 2G12 and the extremely low off-rate constant of PGT121, detected with BG505 SOSIP.664 trimers, are noteworthy.

To understand neutralization, we must measure the affinity of NAbs against optimal antigenic mimics of functional Env trimers, spaced similarly to the spikes on virions. Our current SPR method partly meets those criteria. Hence, if NAbs with similar affinities turn out to protect to widely different extents, validly determined kinetics and stoichiometry of NAb binding might explain why. The SPR-based measurements we describe here complement affinity measured by ITC and stoichiometry determined both by ITC and EM.

For a vaccine to work, its resulting NAb response must reduce the residual infectivity of the inoculum to such a low level that the infection aborts. But how relevant the PF measured *in vitro* is to protection *in vivo* will depend on the design of the neutralization assay. For example, 2G12 neutralizes partly by decelerating entry after viral attachment to target cells, whereas CD4bs-, V3-, and MPER-directed NAbs to various extents shorten the infectious half-life of virions suspended in fluid phase [78,79]. How long virus and NAb are incubated before they reach the target cells will affect which mode of neutralization dominates, and probably how the kinetics of NAb binding influence the PF. If NAbs, virus, and cells are all mixed simultaneously, the on-rate constant might dominate, but the off-rate constant will remain important in the dynamic competition between NAbs and receptors, and between productive entry and abortive pathways [78,80].

NAbs are also being considered for use in therapeutic passive immunization aiming to control or even clear chronic HIV-1 infection [4,73,81-83]. When NAbs are administered directly, those with the most favorable binding and neutralizing properties could be selected and combined. Selection criteria might include complementary kinetic profiles and binding properties maximizing occupancy of NAbs on virus. The latter will depend on the stoichiometry of the binding of the individual NAbs, as well as any synergy or cooperativity among them [84].

Lastly, the kinetics of NAb binding may also inform immunogen design. The exposure of an epitope is likely to be reflected in the on-rate constant for the corresponding NAb. Thus, engineered mutants of native-like trimers that bind the same NAb with different kinetics, in particular with distinct on-rates, might be compared as experimental immunogens in the search for strong inducers of bNAb responses.

## Methods

### Design of Env constructs

The BG505 *env* gene (BG505.W6M.ENV.C2, GenBank accession numbers ABA61516 and DQ208458) is derived from a neonatal subtype A HIV-1 founder virus [85]. BG505 SOSIP.664 gp140 was constructed by introducing several sequence modifications (all numbering is based on the HxB2 sequence): A501C and T605C, to create a disulfide bond between gp120 and gp41_ECTO_[7]); I559P in gp41_ECTO_, to increase trimer stability [13]; REKR (HXB2 Env amino-acid residues 508–511) changed to RRRRRR (R6) at the cleavage site between gp120 and gp41_ECTO_, to promote proteolytic processing by furin [8]); T332N in gp120, to allow the binding of bNAbs that depend on glycan-332 [58]; a stop codon at gp41_ECTO_ residue 664, to improve trimer solubility and homogeneity [11,86]). The codon-optimized gene for BG505 SOSIP.664 gp140 was produced by Genscript (Piscataway, NJ) and cloned into the vector pPPI4 after digestion with *PstI* and *NotI*[7]. Neutralization of the sequence-matched BG505.T332N pseudovirus, reported elsewhere for IgG [29], was performed here with Fabs in the same Tzm-bl assay.

The same trimers were also engineered to contain His- or D7324-epitope-tags at the C-terminus of gp41_ECTO_, by inserting the amino-acid sequences GSGSGGSGHHHHHHHH or GSAPTKAKRRVVQREKR, respectively, between residue 664 in gp41_ECTO_ and the stop codon [17].

The monomeric BG505 gp120 construct was created by introducing a stop codon into the SOSIP.664 gp140 gene at residue 512; the cleavage site was reverted to wild-type (REKR); C501 was reverted to A501; and the L111A substitution was introduced to prevent gp120 dimerization [45,87]. Furthermore, to allow capture by antibody D7324, substitutions R500K and G507Q were introduced into the C5 region. With these changes, the C-terminal twelve residues of our BG505 gp120 protein are KAKRRVVQREKR.

To study the effects of cleavage and the SOSIP modifications, the BG505 SOSIP.664 gp140, referred to previously as SOSIP.R6 for simplicity and comparative purposes [23], was compared with five other previously described constructs, all six Env proteins containing the D7324-tag C-terminal to residue 664 [23]. SOS.R6 is fully cleaved, contains the intermolecular SOS bond but lacks the I559P modification; *WT.SEKS* has the natural REKR cleavage site replaced by the non-scissile motif *SEKS*, but lacks the SOS and I559P changes; *SOS.SEKS* has the non-scissile motif, contains the SOS change but lacks I559P; *IP.SEKS* has the non-scissile motif, contains the I559P change but lacks SOS; *SOSIP.SEKS* has the non-scissile motif, and contains the SOS and I559P changes. Note that the four uncleaved Env proteins have designations in italics. The IP.R6 construct that is fully cleaved, contains the I559P change but lacks the intermolecular SOS bond; this construct and WT.R6 were not studied here as their subunits dissociate [23]. The various Env proteins were all expressed in HEK293T cells and purified by 2G12-affinity followed by size-exclusion chromatography (SEC), as described previously [17]. At the SEC purification stage, the SOSIP.664 trimers were separated from the monomeric gp120-gp41_ECTO_ protomers (Additional file 1: Figure S1). Thus, the protomer is stabilized by the SOS disulfide bond and also contains the IP change. The trimer and protomer fractions used were deemed >95% pure.

### Antibodies

VRC01 and PGV04 (also named VRC PG04) to the CD4bs [59,88] were provided by John Mascola (Vaccine Research Center, NIH); 14e and 19b, both V3-directed [17,89], by James Robinson (Tulane University); b12 and b6, both to the CD4bs [90,91]; F240 to cluster I in gp41 [92]; 2G12 to a mannose-dependent epitope [57], PGT121, PGT122, PGT123, and PGT128, to glycan- and V3-base-dependent epitopes [43]; and PG9, PG16, and PGT145 to V1V2- and glycan-dependent quaternary-structural epitopes [16,44] were supplied by The Scripps Research Institute and the International AIDS Vaccine Initiative’s reagent repository. PGT151 to a novel epitope spanning gp120 and gp41_ECTO_ was supplied by The Scripps Research Institute [46,47]. Fabs of PGV04, PGT123, and PGT145 were expressed and purified as previously described [93]; PGT145 Fab was co-expressed with the tyrosine-sulfating TPST-1 enzyme. Then, after ion exchange purification, the fraction with the highest degree of tyrosine sulfation, as determined by mass spectrogram, was selected [93].

### Surface plasmon resonance

All experiments were performed at 25°C on a Biacore 3000 instrument (GE Healthcare). We used three different methods for immobilizing Env.

In the first method, epitope-tagged Env was captured by the polyclonal, affinity-purified Ab preparation D7324 (Aalto BioReagents, Dublin, Eire). This method was used for qualitative comparison of Env constructs (Figures 1 and 2). First, D7324 was covalently coupled to the dextran on CM5 chips. During coupling and capture steps, the flow rate was 10 μl/min. The surface of the chip was activated by injecting NHS and EDC (1:1 [v/v] mixture of N-hydroxysuccinimide/n-ethyl-N’-(3-diethylaminopropyl) carbodiimide) for 7 min. D7324, diluted to 50 μg/ml in immobilization buffer (10 mM sodium acetate pH 4.5), was injected for 7 min, yielding ~7000 RU. After the Ab coupling, ethanolamine was injected for 7 min to deactivate the surface. Epitope-tagged Env, diluted in running buffer to 20 μg/ml, was then captured, giving ligand immobilization levels, i.e., R_L_ values, of ~500 RU (or ~ 425 RU, i.e., 15% lower for gp120 to achieve equal amounts (mol) on the surface). Channels with D7324 Ab but no Env served as controls. Flow rates of 50 μl/min were used for all Ab binding, in order to minimize mass-transport limitation. After the binding of each MAb, the surface was regenerated by a 90-s pulse of 10 mM glycine, pH 2.0, at a flow rate of 75 μl/min, which allows the coupled D7324 to be reused with a new batch of Env. Some drift, i.e. dissociation of ligand from the capturing Ab, occurred. Hence, even with only marginal experimental error and changes in drift during a cycle, this process cannot be perfectly controlled for by 0-analyte subtraction. Thus, for the qualitative evaluations based on D7324-epitope tagged constructs, only the background of the control channel was subtracted, because of the inconstant dissociation of ligand (drift) inherent to that method. The mean drift was 1.2 ^**.**^ 10^−3^ - 1.3 ^**.**^ 10^−2^ (RU/s) for the trimers and 1.0 ^**.**^ 10^−2^ - 1.6 ^**.**^ 10^−2^ (RU/s) for the monomers in Figures 1 and 2. This shortcoming necessitated the use of more stable immobilization methods for modeling the kinetics of binding, particularly when the MAb-Env dissociation was extremely slow.

In the second immobilization method, His-tagged forms of Env were captured on Ni^2+^-NTA chips. All Abs (IgG or Fab) were screened for non-specific binding to Ni^2+^ by comparing Ni^2+^-loaded and NTA-only channels. Several Abs did give high backgrounds and could only be studied by other approaches. Despite this limitation, the Ni^2+^-based method was used whenever possible, because it gave the most stable immobilization of Env thereby promoting high-quality kinetic modeling. This was particularly so for gp120-gp41_ECTO_ protomers. This His-tag immobilization was used for full kinetic analysis of all IgGs and Fabs with no detectable background binding to Ni^2+^-NTA, specifically 2G12 IgG, PGT121 IgG, PGT122 IgG and Fab, PGT123 Fab, VRC01 IgG and Fab, and PG16 IgG. After metallic contaminants had been removed by a pulse of EDTA (350 mM) in running buffer (150 mM NaCl, 10 mM HEPES, pH 7.4 plus 0.005% Tween20) for 1 min at a flow rate of 30 μl/min, the chip was loaded with Ni^2+^ by injecting NiCl_2_ at 2.5 mM for 1 min with a flow rate of 5 μl/min, yielding an addition of ~50 RU. The running buffer was supplemented with 50 μM EDTA to minimize non-specific binding. His-tagged Env at 10 μg/ml was injected at 5 μl/min for 2–3 min to capture again an amount yielding a signal of ~500 RU (=*R*_*L*_, see Additional file 2: Table S1). The analyte, whether IgG or Fab, was injected into the Env and control channels at a flow rate of 50 μl/min at concentrations titrated down from 0.5 or 1 μM until no significant signal was obtained. Association was recorded for 300 s and dissociation for 600 s in standard experiments, but longer dissociation times (1200 s) were sometimes used in attempts to quantify very low off-rate constants. After the binding of each Ab, the NTA-chip surface was regenerated with a pulse of EDTA (350 mM) for 1 min at a flow rate of 30 μl/min, followed by 3 washes with running buffer (containing 50 μM EDTA).

The third method for immobilizing Env was used for Abs that bound detectably to Ni^2+^. In these cases, His-tagged trimers were instead captured by an immobilized anti-His Ab (GE Healthcare), which was coupled to CM5 chips as for Ab D7324 (see above), to yield ~15000 RU. Abs were then injected at a flow rate of 50 μl/min at concentrations titrated downwards from 0.5 or 1 μM until insignificant binding was reached. After each cycle of Ab binding, we regenerated the anti-His surface by injecting a single pulse of 10 mM Glycine (pH 1.5) for 60 s at a flow rate of 30 μl/min. This approach, which immobilizes Env at stabilities intermediate between Methods 1 and 2 above, was used for PGV04 (both IgG and Fab), and for IgG versions of PGT123, 2G12, PG9, PGT145, and PGT151. The 2G12 IgG was also tested by using the Ni-NTA capture method for cross-validation. We found substantial background binding for sCD4 with both Ni-NTA and anti-His antibody; while that background does not preclude the qualitative assessment of sCD4 binding to SOSIP.664 trimers [17], it precludes rigorous kinetic analysis. Accordingly, we chose not to study sCD4 binding by any of the SPR methods here.

In addition to immobilizing Env, we used the untagged trimer or protomer as analyte. The Abs 14e, 19b, and PGT145 were captured to ~ 550 RU onto the chip by anti-Fc Ab, immobilized the same way as the D7324 Ab above, and the binding of untagged BG505 SOSIP.664 trimers or protomers titrated down from 200 nM and at a flow rate of 50 μl/min was monitored. Association was recorded for 300 s, dissociation for 600 s.

### Evaluation of binding data

For all kinetic modeling, and hence with all data derived from His-tagged constructs, background values from control channels, as well as those obtained by injecting buffer (0 analyte) in the test channel, were subtracted. In these experiments, the analytes were titrated to allow for a complete modeling of the kinetics. Other experiments were performed at single analyte concentrations to assess differential antigenicity qualitatively.

To minimize the risk of mass-transfer limitation, all experiments were performed with a flow rate of 50 μl/ml. Preliminary experiments showed no increase in signal when the rate was > 40 μl/ml. Furthermore, the data were scrutinized for possible mass-transport limitation by the following criteria before further modeling: first, the ln(dR/dt) plots were inspected and found to be approximately straight with a downward slope in the association phase. Second, the tentatively best model with a mass-transport component added was fitted globally to ascertain that the resulting *k*_*t*_ values were > 10^8^ (RU M^−1^ s^−1^). These *k*_*t*_ values were typically either just above 10^8^ (RU M^−1^ s^−1^) with T(*k*_*t*_) > 10, or they were > > 10^8^ (RU M^−1^ s^−1^) with T(*k*_*t*_) < 10; both outcomes were deemed to be acceptable. Third, as a further precaution, in the final modeling (without any mass-transfer component), only fitted parameters with T > 10 were accepted. It should also be noted that the majority of the *k*_*on*_ values measured were < 10^5^ (M^−1^ s^−1^), i.e. below the risk zone for serious mass transfer limitations.

Two different kinetic models were applied (as included in BIAevaluation version 4, GE Healthcare). For IgG molecules known to be capable of bivalent binding, the bivalent model yielded variable improvements in fit over a simple Langmuir model, see Additional file 3: Table S2). Monovalent analytes (Fabs, 2G12, and trimer against immobilized PGT145) were fitted with the simple Langmuir model.

Data on neutralization of BG505.T332N pseudovirus by IgG NAbs from previous studies [17,47] and in addition by Fabs, obtained by the same method, were converted to logarithmic relative infectivities and modeled with an unconstrained sigmoid function with a variable slope, to allow us to identify the persistent fractions, PF [62-64]. We also determined the Hill slope and IC_50_ values by fitting a regular sigmoid function with variable slope and top and bottom plateaus constrained to 1 and 0, respectively. Statistical analyses are described in the SI.

## Competing interests

JPJ, RWS, ABW, IAW, JPM, AC, and PJK are listed on a patent application relating to the general use of BG505 SOSIP.664 gp140 trimers. The other authors have no competing interests.

## Authors’ contributions

AY performed the SPR experiments and modeling under the guidance of PJK. RR and AC produced and purified the Env proteins. RD performed the neutralization assays. JPJ, DRB, ABW, and IAW provided key reagents. RWS and JPM designed the protein constructs. PJK designed the experiments and analyzed the data with input from AY, JPJ, ABW, and IAW. PJK wrote the paper with input from AY, JPJ , ABW, IAW, RWS, and JPM. All authors read and approved the final manuscript.

## Supplementary Material

Additional file 1 **BN-PAGE analysis of BG505 SOSIP.664 trimer and gp120-gp41ECTO protomer. Figure S1.** BN-PAGE analysis of BG505 SOSIP.664 trimer and gp120-gp41ECTO protomer. **Figure S2.** gp120-gp41ECTO protomer and gp120 monomer binding to immobilized V3 antibodies. **Figure S3.** Model components of bivalent IgG interaction with SOSIP.664 trimers and gp120-gp41ECTO protomers. **Figure S4.** The effect of variation in ligand density on the degree of bivalent binding. **Figure S5.** Fab binding to SOSIP.664 trimers and gp120-gp41ECTO protomers; and trimer binding to immobilized PGT145. Click here for file

Additional file 2: Table S1 Analysis of variation in amounts of immobilized ligand. Click here for file

Additional file 3: Table S2 Comparison of Langmuir and bivalent model fits to IgG binding. **Table S3.** Fab and other monovalent interactions with SOSIP.664 trimers and gp120-gp41ECTO protomers. **Table S4.** Comparison of *Sm* estimates. **Table S5.** Bivalent modeling of IgG binding to BG505 SOSIP.664 trimer and gp120-gp41ECTO protomer. **Table S6.** Neutralization of BG505.T332N by Fabs. Click here for file

Additional file 4 Supplementary results and commentary. Click here for file
