# Supplementary material for: Differential binding of neutralizing and non-neutralizing antibodies to native-like soluble HIV-1 Env trimers, uncleaved Env proteins, and monomeric subunits
Source: Retrovirology. 2014 May 29;11:41. doi: 10.1186/1742-4690-11-41 (PMC4067080; doi:10.1186/1742-4690-11-41)
Supplement: Additional file 1 — BN-PAGE analysis of BG505 SOSIP.664 trimer and gp120-gp41ECTO protomer. Figure S1. BN-PAGE analysis of BG505 SOSIP.664 trimer and gp120-gp41ECTO protomer. Figure S2. gp120-gp41ECTO protomer and gp120 monomer binding to immobilized V3 antibodies. Figure S3. Model components of bivalent IgG interaction with SOSIP.664 trimers and gp120-gp41ECTO protomers. Figure S4. The effect of variation in ligand density on the degree of bivalent binding. Figure S5. Fab binding to SOSIP.664 trimers and gp120-gp41ECTO protomers; and trimer binding to immobilized PGT145. [file 1742-4690-11-41-S1.pdf]

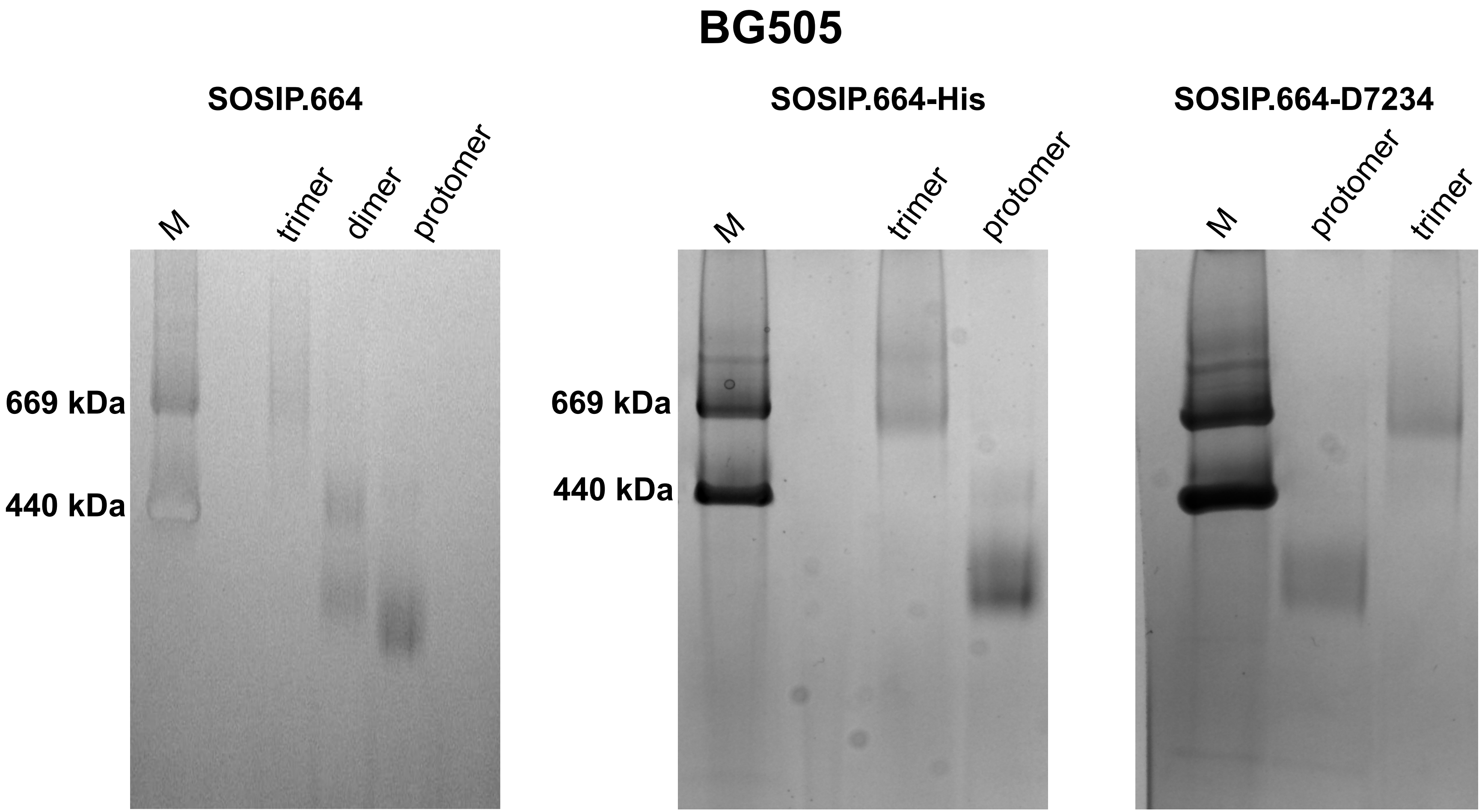

Figure S1. BN-PAGE analysis of BG505 SOSIP.664 trimer and gp120-gp41$_{ECTO}$ protomer. BG505 SOSIP.664 protein was first purified by 2G12-affinity chromatography and thereafter by SEC, which separates the trimer, dimer, and the monomeric gp120-gp41$_{ECTO}$ protomers as indicated on the left-hand gel. The left-hand gel shows untagged trimer and protomer, the middle one His-tagged, and the right-hand one D7324-epitope-tagged trimer and protomer. The proteins were stained with Commassie blue. The molecular-mass markers (M), thyroglobulin and ferritin, are the same for all three gels.

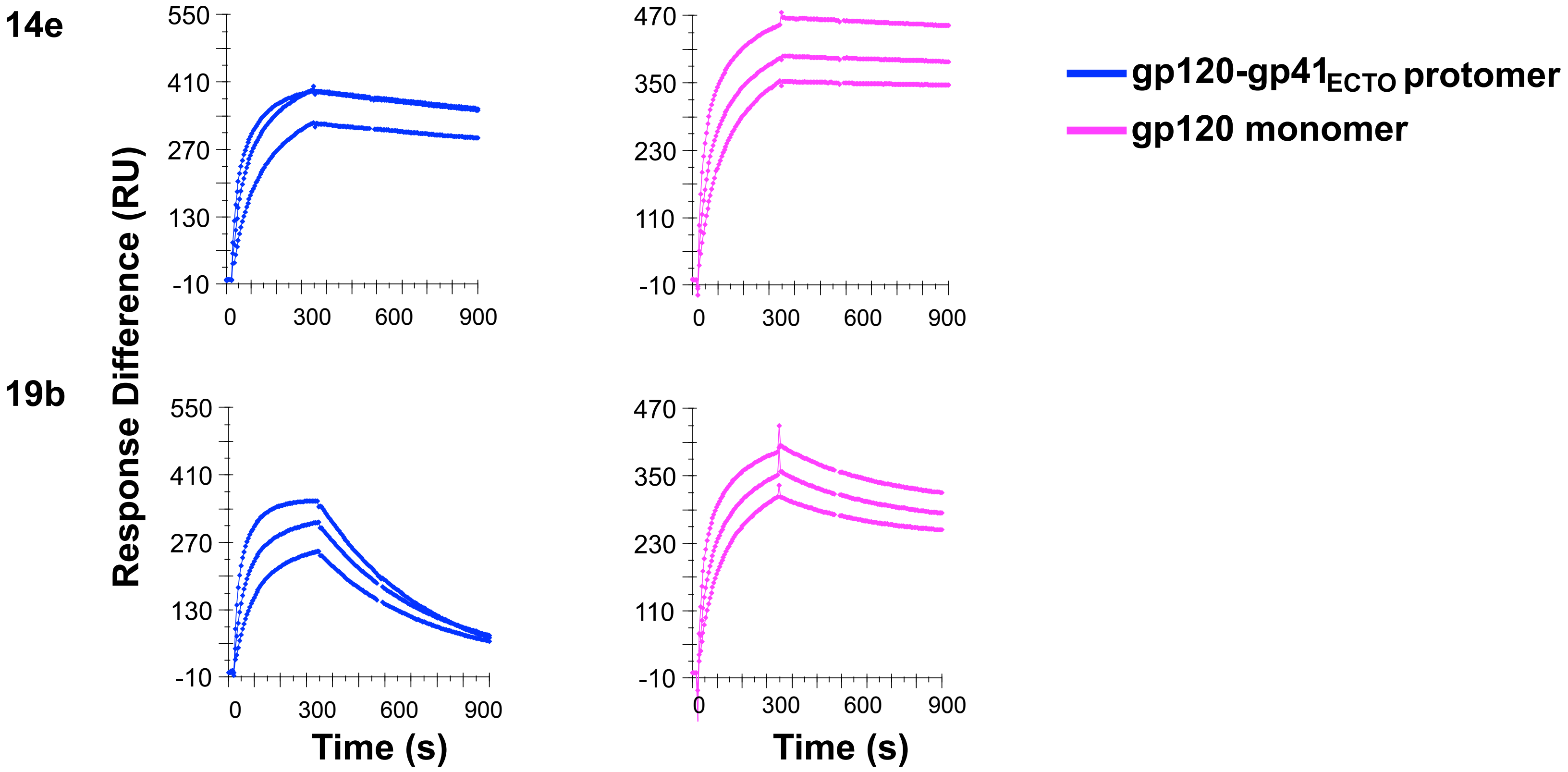

Figure S2. gp120-gp41$_{ECTO}$ protomer and gp120 monomer binding to immobilized V3 antibodies. The sensorgrams show the binding of untagged soluble BG505 gp120-gp41$_{ECTO}$ protomer (left) and gp120 monomer (right) at 500, 250, and 125 nM to the anti-Fc-immobilized V3-specific non-NAbs 14e (top) and 19b (bottom). The lengths of the axes have been adjusted to control for the difference in mass and thereby signal between gp120 monomer and gp120-gp41$_{ECTO}$ protomer, so that horizontal lines crossing the two y axes perpendicularly would represent the same degree of binding (in moles) in the sensorgrams.

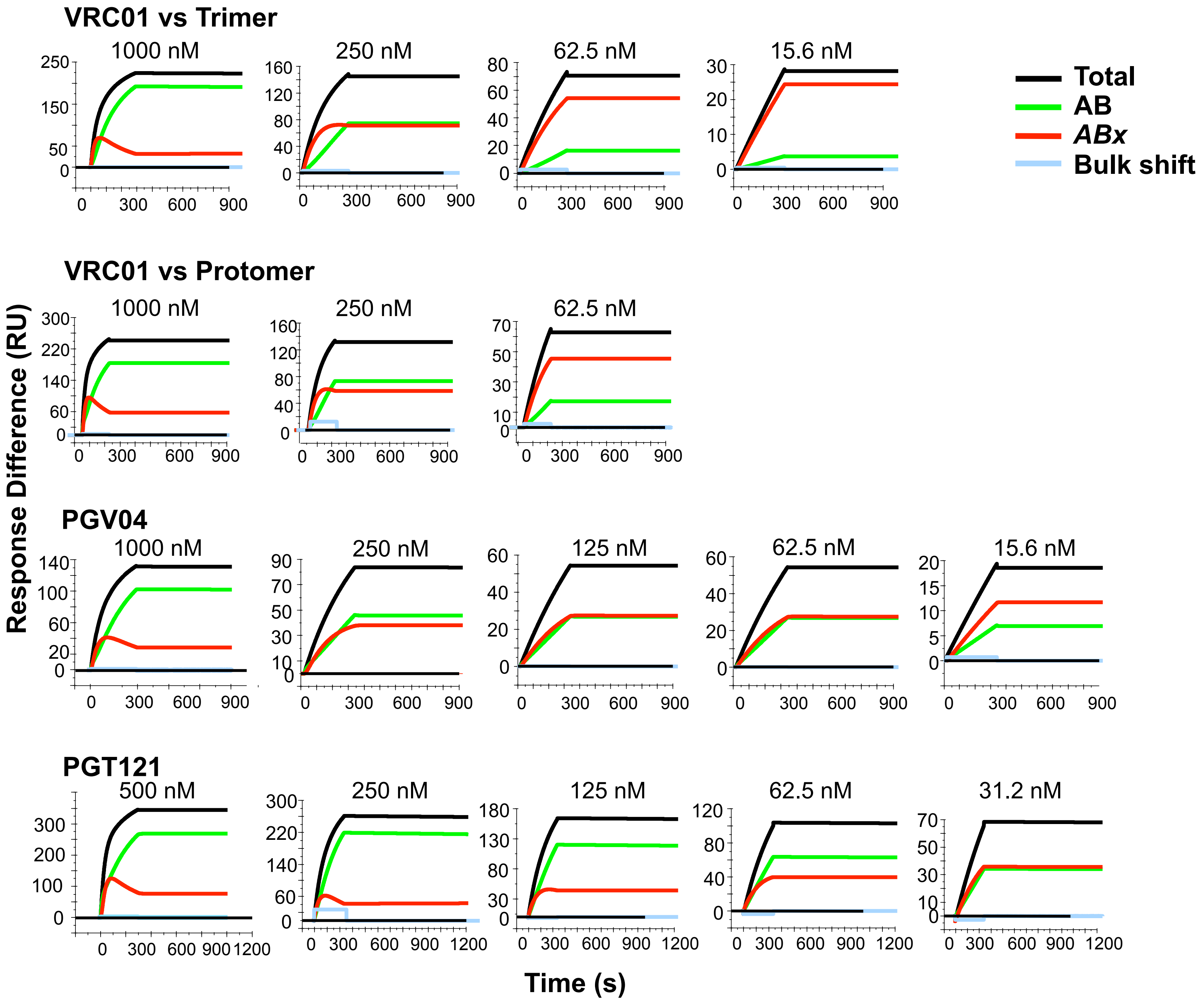
VRC01 vs Trimer
1000 nM
250 nM
62.5 nM
15.6 nM
Total
AB
ABx
Bulk shift
VRC01 vs Protomer
1000 nM
250 nM
62.5 nM
PGV04
1000 nM
250 nM
125 nM
62.5 nM
15.6 nM
PGT121
500 nM
250 nM
125 nM
62.5 nM
31.2 nM
Response Difference (RU)
Time (s)

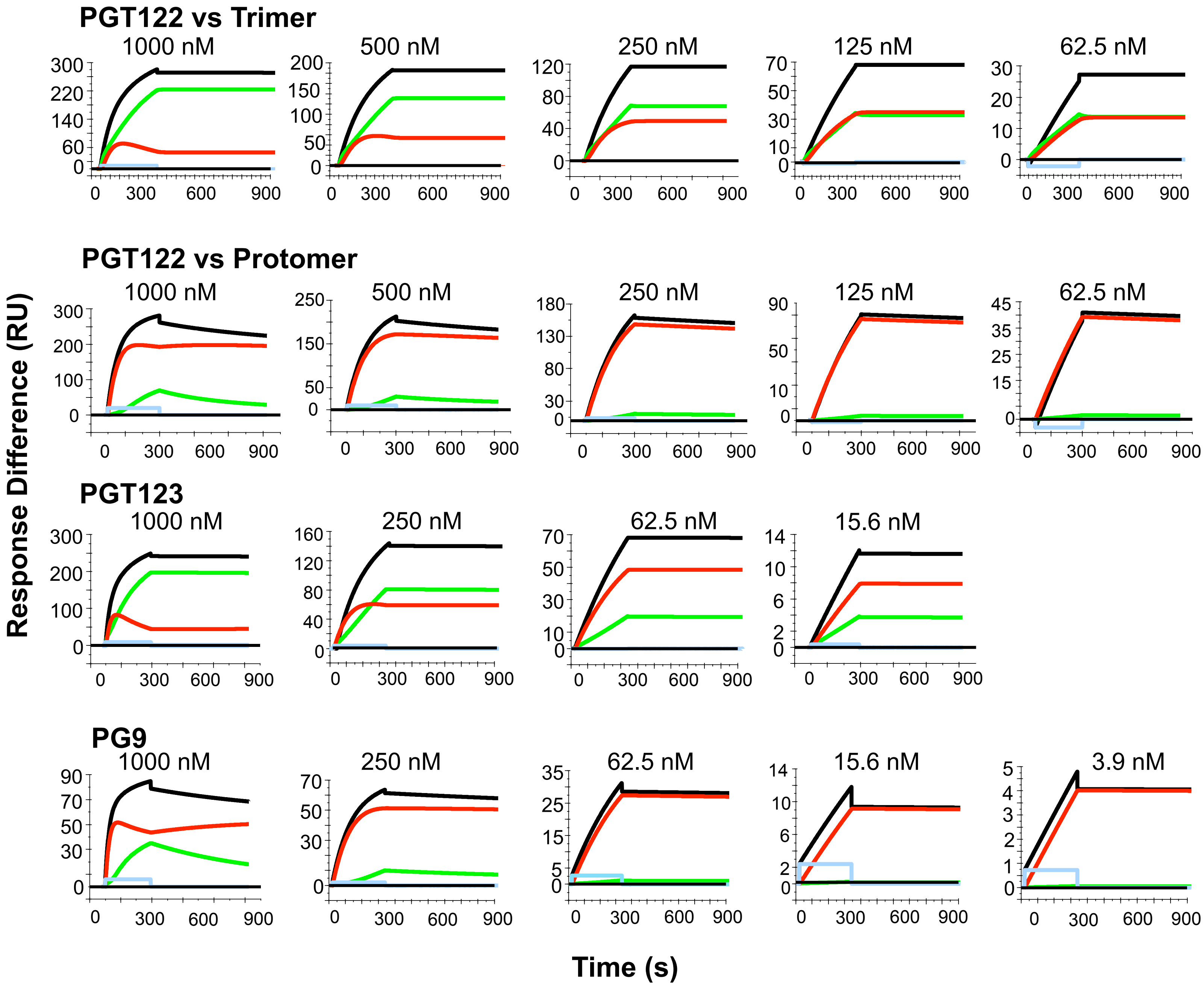

PGT122 vs Trimer
1000 nM
500 nM
250 nM
125 nM
62.5 nM
PGT122 vs Protomer
1000 nM
500 nM
250 nM
125 nM
62.5 nM
PGT123
1000 nM
250 nM
62.5 nM
15.6 nM
PG9
1000 nM
250 nM
62.5 nM
15.6 nM
3.9 nM
Response Difference (RU)
Time (s)

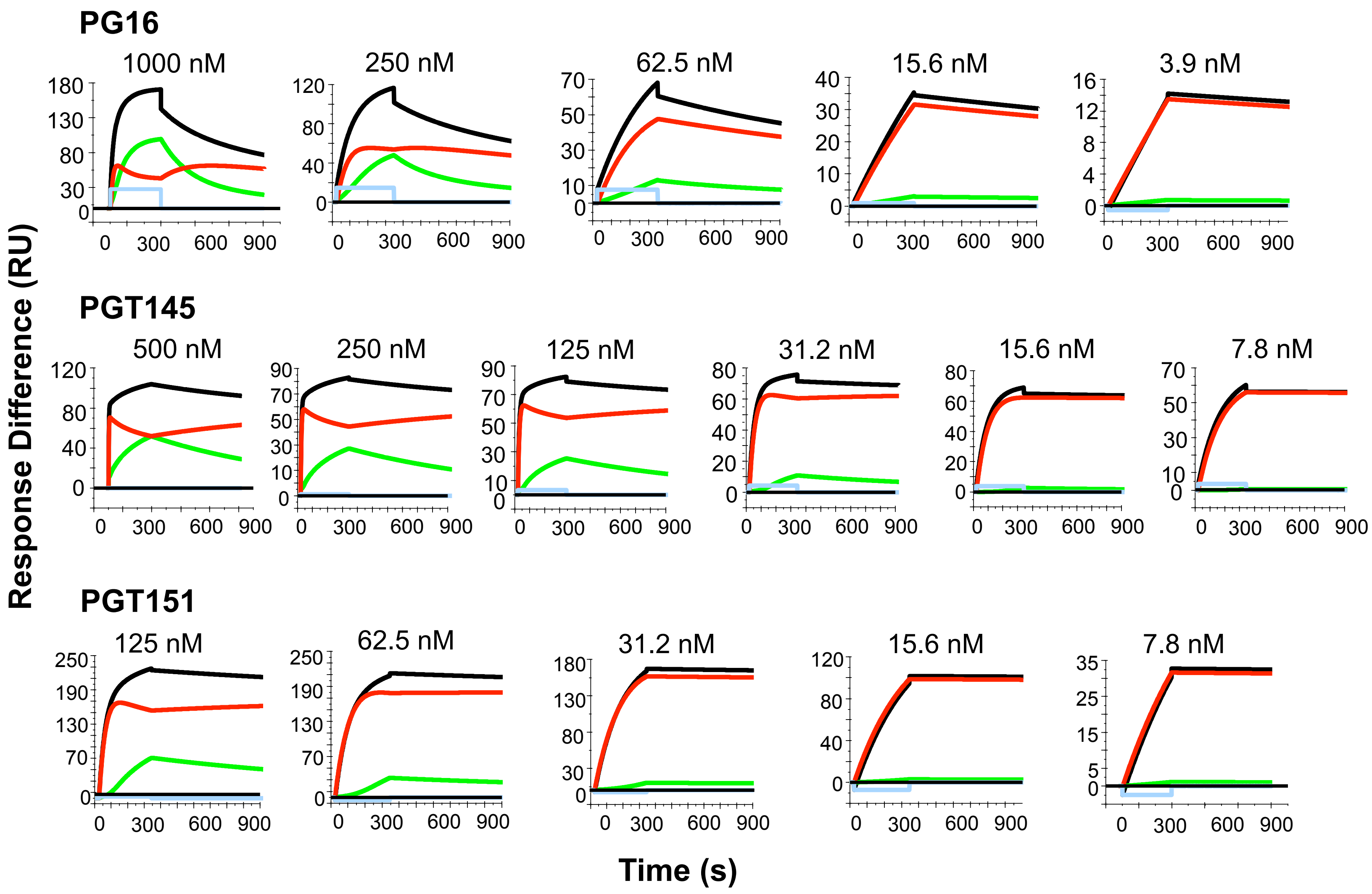

Figure S3. Model components of bivalent IgG interaction with SOSIP.664 trimers and gp120-gp41$_{ECTO}$ protomers. The diagrams show the components of the bivalent model (as indicated in the legend to the right) for the full titration ranges. The initial monovalent interaction, denoted AB, is followed by a bivalent strengthening of the interaction, i.e. the epitope ligation by the second paratope of the IgG molecule, denoted ABx. Small residual bulk shifts (vertical parts of the curve for the total reaction) sometimes have to be corrected for in spite of the control channel subtraction. No correction was needed for drift, i.e. ligand dissociation. Note that the bivalent binding often increases with decreasing concentration.

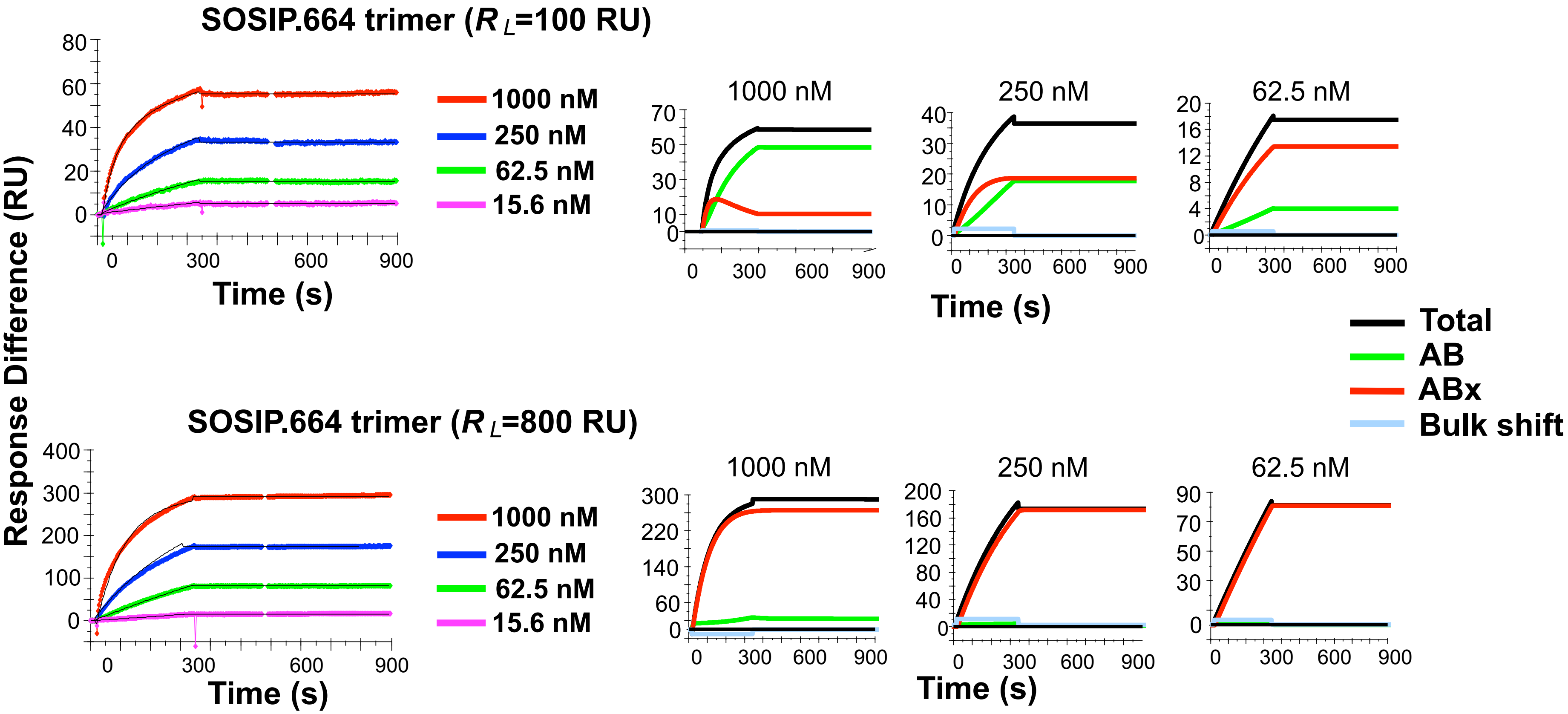

Figure S4. The effect of variation in ligand density on the degree of bivalent binding. BG505 SOSIP.664 trimer was captured to two different levels, R_L = 100 RU and 800 RU, i.e., below and above the standard R_L = 500 RU. The diagrams show the components of the bivalent model (as indicated in the legend to the right) for three concentrations of analyte. At 800 RU the modeled binding by VRC01 was exclusively bivalent. At 100 RU the bivalent component was ~ 15 % at 1000 nM and increased to represent the majority at 62.5 nM.

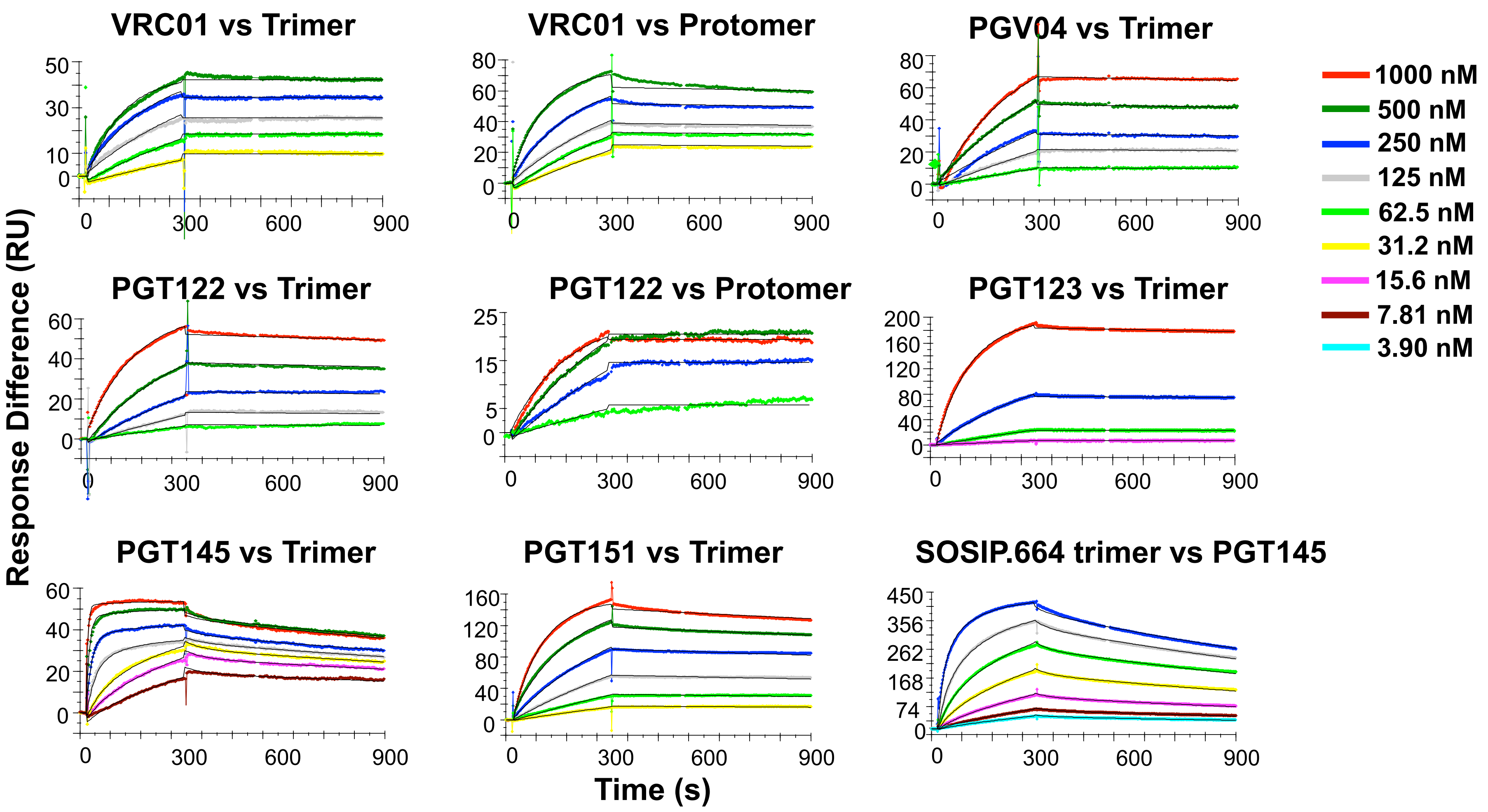

Figure S5. Fab binding to SOSIP.664 trimers and gp120-gp41$_{ECTO}$ protomers; and trimer binding to immobilized PGT145. All sensorgrams represent monovalent interactions; all show Fabs of Nabs binding to trimer or protomer as indicated, except the last to the right in the bottom row, which shows trimer as analyte binding to immobilized PGT145 IgG. The empirical curves are color-coded according to concentration as indicated in the legend to the upper right. Note that the concentration ranges differ among the diagrams. Also note that the scales of the y axes differ; thus VRC01 Fab binding to protomer is stronger than to trimer; and PGT122 Fab binding to trimer is stronger than to protomer. The sensorgrams show one of the replicates (n) given in Table S3. In some experiments the dissociation phase had to be extended to 20 min (not shown) to achieve significant values (T>10) for k_{off} ; in some cases k_{off} was still insignificant (see Table S3).
