# Supplementary material for: Differential binding of neutralizing and non-neutralizing antibodies to native-like soluble HIV-1 Env trimers, uncleaved Env proteins, and monomeric subunits
Source: Retrovirology. 2014 May 29;11:41. doi: 10.1186/1742-4690-11-41 (PMC4067080; doi:10.1186/1742-4690-11-41)
Supplement: Additional file 2: Table S1 — Analysis of variation in amounts of immobilized ligand. [file 1742-4690-11-41-S2.pdf]

Supplementary Tables

Table S1. Analysis of variation in amounts of immobilized ligand

	D7324									NTA-Ni^{2+}		Anti-His
**Statistic**	**Trimer**	**Protomer**	**gp120** [d]	**SOSIP.R6**	**SOS.R6**	***SOSIP.SEKS***	***IP.SEKS***	***SOS.SEKS***	***WT.SEKS***	**Trimer**	**Protomer**	**Trimer** [e]
**n** [a]	21	21	21	16	16	16	16	16	16	104	10	71
**R_L (RU)** [b]	509	504	426	523	502	526	507	504	519	529	519	400
**SD**	44.4	51.1	65.6	61.3	21.7	51.5	28.0	25.2	50.0	31.9	4.78	48.0
**SD %** [c]	8.7	10	15	12	4.3	9.8	5.5	5.0	9.6	6.0	0.92	12
**95% CI**	489-529	480-527	396-456	491-556	490-513	498-553	493-522	490-517	492-545	523-535	515-522	389-411

[a] n=the number of experiments for each form of Env and type of immobilization. Experiments not included here are those that used non-standard Env immobilization levels (Figure S4) and the immobilization of NAb PGT145 with trimer as analyte (Table S3 and Figure S5).

[b] The mean R_L values for the immobilized forms of Env measured in RU are given.

[c] The standard deviation as a percentage of the mean is given with two significant digits, other data with three.

[d] Since the mass of gp120 is ~ 86% that of the protomer, a correspondingly lower immobilization level measured in RU was aimed for in order to keep the amount of gp120 constant for the immobilization of the three forms of Env. An immobilization level of 84% for gp120 relative to those of trimer and protomer, i.e. close to the ideal was achieved.

[e] Somewhat lower immobilization of trimer was achieved by the anti-His method than the others, although the ligand dissociation was more limited than for D7324 capture. Since the anti-His immobilization was not used for comparing different forms of Env, these experiments with somewhat lower R_L were only compared within the set.
