# Supplementary material for: Differential binding of neutralizing and non-neutralizing antibodies to native-like soluble HIV-1 Env trimers, uncleaved Env proteins, and monomeric subunits
Source: Retrovirology. 2014 May 29;11:41. doi: 10.1186/1742-4690-11-41 (PMC4067080; doi:10.1186/1742-4690-11-41)
Supplement: Additional file 3: Table S2 — Comparison of Langmuir and bivalent model fits to IgG binding. Table S3. Fab and other monovalent interactions with SOSIP.664 trimers and gp120-gp41ECTO protomers. Table S4. Comparison of Sm estimates. Table S5. Bivalent modeling of IgG binding to BG505 SOSIP.664 trimer and gp120-gp41ECTO protomer. Table S6. Neutralization of BG505.T332N by Fabs. [file 1742-4690-11-41-S3.pdf]

Table S2. Comparison of Langmuir and bivalent model fits to IgG binding

NAb (IgG)	L [b] χ^2 [c]	B [b] χ^2	L/B [c] χ^2 ratio	L [d] K_d (nM)	B/L [e] K_{d1}/K_d	L [f] T(k_{off})	B [g] T(k_{on2})	B [g] T(k_{off2})
VRC01 (n=3) [a]	7.0 ± 1.8	1.3 ± 0.24	5.7 ± 1.6	0.056 ± 0.044	55 ± 39	1.4 ± 0.94	55 ± 30	64 ± 28
VRC01 (n=2) *protomer*	12 ± 3.5	1.5 ± 0.69	12 ± 7.9	0.073 ± 0.072	80 ± 80	0.71 ± 0.61	27 ± 10	220 ± 180
PGV04 (n=3) [a]	3.0 ± 1.6	1.5 ± 0.73	2.0 ± 0.40	0.0080 ± 0.0010	32 ± 20	0.67 ± 0.42	36 ± 16	260 ± 110
PGT121 (n=3)	1.9 ± 0.55	0.27 ± 0.034	7.5 ± 2.6	0.091 ± 0.048	71 ± 66	7.9 ± 4.7	22 ± 4.9	28 ± 8.1
PGT122 (n=2)	5.1 ± 2.5	1.3 ± 0.55	3.7 ± 0.37	$9.7 \cdot 10^{-4}$ ± $9.6 \cdot 10^{-4}$	$1.3 \cdot 10^{4}$ ± $1.2 \cdot 10^{4}$	0.060 ± 0.060	26 ± 14	430 ± 350
PGT122 (n=2) *protomer*	3.4 ± 1.4	0.82 ± 0.14	4.6 ± 2.5	8.7 ± 0.87	74 ± 22	100 ± 1.2	44 ± 30	28 ± 14
PGT123 (n=2)	5.9 ± 0.29	0.71 ± 0.051	8.4 ± 0.20	0.0082 ±0.0040	160 ±52	0.22 ± 0.11	25 ± 14	37 ± 21
PG9 (n=2)	0.79 ±0.040	0.23 ± 0.0085	3.5 ± 0.047	5.4 ± 0.25	14 ± 1.0	68 ± 4.1	58 ± 44	64 ± 46
PG16 (n=2)	20 ±0.050	5.6 ± 0.015	3.6 ± $6.3 \cdot 10^{-4}$	35 ± $1.8 \cdot 10^{-9}$	5.6 ± 0.19	110 ± 1.9	34 ± 18	36 ± 19
PGT145 (n=2)	3.2 ± 0.52	0.59 ± 0.12	5.9 ± 2.1	0.16 ± 0	18 ± 6.0	54 ± 6.4	12 ± 5.8	17 ± 11
PGT151 (n=2)	3.6 ± 0.18	1.1 ± 0.11	3.3 ± 0.17	0.25 ± 0.0055	25 ± 1.3	37 ± 1.8	6.9 ± 2.0	7.2 ± 2.2

[a] The tabulated values are the means ± s.e.m. of n independent experiments. The ligand was the BG505 SOSIP.664 trimer except in rows marked *protomer*.

[b] L=Langmuir model. B=Bivalent model.

[c] The χ^2 value shows the goodness of the fit of the models, the lower the value the better the fit. The fits of the Langmuir and bivalent models were compared by giving the ratio of the χ^2 value for Langmuir over that for the bivalent model. Ratios >1 indicate superiority of the bivalent fit.

[d] The K_d values were derived by the Langmuir fit and represent an average dissociation constant for the entire IgG molecule, regardless of whether it binds with one or two paratopes. It has the dimension of concentration (nM). The other parameters in the table are dimensionless.

[e] K_d applies to the Langmuir model, K_{d1} to the bivalent model. As a measure of the difference between bivalent and Langmuir models, the K_{d1}/K_d ratios were calculated. Since K_{d1} is the dissociation constant for the monovalent component in the bivalent binding, whereas K_d describes the binding by the whole IgG molecule, the ratio also constitutes an avidity index.

[f] The T value indicates the significance of a parameter value. The k_{off} values obtained by the Langmuir fit were sometimes insignificant, a sign of the inferiority of that modeling.

[g] The T values of the kinetic constants of the second component of the bivalent model, k_{on2} and k_{on2}, are given as measures of the significance of the bivalency.

Table S3. Fab and other monovalent interactions with SOSIP.664 trimers and gp120-gp41$_{ECTO}$ protomers

**Analyte**	**L** [b] k_{on} (1/Ms)	**L** [b] k_{off} (1/s)	**L** [b] K_d (nM)	**B/L** [c] k_{on1}/k_{on}	**B/L** [c] k_{off1}/k_{off}	**B/L** [c] K_{d1}/K_d
VRC01 Fab (n=1) [a]	$1.4 \cdot 10^4$	$4.9 \cdot 10^{-5}$	3.4	0.52	0.10	0.21
VRC01 Fab (n=1) *protomer*	$4.6 \cdot 10^4$	$< 10^{-4}$	< 1	0.87	ND [d]	ND
PGV04 Fab (n=2)	$4.6 \cdot 10^3$ $\pm 1.6 \cdot 10^2$	$3.4 \cdot 10^{-5}$	7.7	0.86	<0.29	<0.77
PGT122 Fab (n=1)	$8.7 \cdot 10^3$	$< 10^{-4}$	< 10	0.53	ND	ND
PGT122 Fab (n=1) *protomer*	$6.0 \cdot 10^3$	$< 10^{-5}$	< 1	0.80	ND	ND
PGT123 Fab (n=3)	$9.7 \cdot 10^3$ ± 42	$5.0 \cdot 10^{-5}$ $\pm 5.0 \cdot 10^{-5}$	5.1 ± 0.62	0.88	0.19	0.22
PGT145 Fab (n=2)	$2.1 \cdot 10^5$ $\pm 9.7 \cdot 10^3$	$4.2 \cdot 10^{-4}$ $\pm 7.8 \cdot 10^{-6}$	2.0 ± 0.088	1.1	1.6	1.4
Trimer (n=3) *PGT145*	$1.0 \cdot 10^5$ $\pm 2.0 \cdot 10^3$	$6.2 \cdot 10^{-4}$ $\pm 6.1 \cdot 10^{-6}$	6.2 ± 0.17	2.4	1.1	0.46
PGT151 Fab (n=2)	$1.4 \cdot 10^4$ ± 50	$1.0 \cdot 10^{-4}$ $\pm 1.0 \cdot 10^{-6}$	7.2 ± 0.060	3.9	3.4	0.88
2G12 (n=5)	$6.1 \cdot 10^4$ $\pm 3.6 \cdot 10^3$	$1.5 \cdot 10^{-4}$ $\pm 2.0 \cdot 10^{-6}$	1.3 ± 0.11	-	-	-

[a] All tabulated values are the means ± s.e.m. of n independent experiments. The ligand is the BG505 SOSIP.664 trimer except in rows marked *protomer* and *PGT145*.

[b] The kinetic constants k_{on} and k_{off} obtained by Langmuir modeling and their ratio, $k_{off} / k_{on} = K_d$ describe the monovalent binding of the Fabs to the SOSPI.664 trimer.

[c] The Langmuir-modeled monovalent Fab binding was compared with the monovalent component of the bivalent modeling of the corresponding IgG, by the calculation of the k_{on1} / k_{on}, k_{off1} / k_{off}, and K_{d1} / K_d ratios.

[d] ND= not determined. These ratios were not considered meaningful when the denominators were unlimited downwards.

Table S4. Comparison of S_m estimates

NAb (IgG)	S_m T_{max} [b]	T_{max} [b]	S_m C_{max} [c]	*T at* C_{max} [c]	S_m *global* R_{max} [d]	χ^2 *global* R_{max} [d]	*T global* R_{max} [d]
VRC01 (n=2)[a]	1.6 ± 0.13	230 ± 100	1.6 ± 0.13	230 ± 100	1.9 ± 0.50	22 ± 6.4	140 ± 13
VRC01 (n=2) *protomer*	0.72 ± 0.18	140 ± 27	0.50 ± 0.0012	130 ± 32	0.52 ± 0.028	64 ± 37	280 ± 199
PGV04 (n=3)	1.5 ± 0.22	530 ± 400	1.9 ± 0.047	400 ± 280	2.8 ± 0.38	15 ± 9.5	300 ± 170
PGV04 Fab (n=3)	1.8 ± 0.018	140 ± 20	1.8 ± 0.018	140 ± 20	1.7 ± 0.19	2.0 ± 0.39	530 ± 82
PGT121 (n=3)	1.6 ± 0.055	600 ± 300	1.7 ± 0.12	590 ± 330	2.8 ± 0.27	66 ± 15	340 ± 140
PGT122 (n=2)	1.6 ± 0.078	190 ± 81	2.0 ± 0.35	160 ± 76	2.5 ± 0.032	26 ± 7.7	140 ± 55
PGT122 (n=2) *protomer*	0.82 ± 0.0020	390 ± 78	0.80 ± 0.024	380 ± 86	0.73 ± 0.063	27 ± 14	120 ± 27
PGT123 (n=2)	2.0 ± 0.071	190 ± 18	1.9 ± 0.039	180 ± 24	2.0 ± 0.0075	28 ± 1.2	170 ± 38
2G12 (n=5)	2.9 ± 0.11	2400 ± 460	2.9 ± 0.11	2400 ± 460	2.9 ± 0.095	110 ± 44	1000 ± 160
PG9 (n=2)	0.97 ± 0.0051	920 ± 350	1.0 ± 0.0043	550 ± 260	1.0 ± 0.018	0.88 ± 0.076	500 ± 21
PG16 (n=2)	0.96 ± 0.022	180 ± 60	1.1 ± 0.030	190 ± 2	0.82 ± 0.018	27 ± 3.0	110 ± 16
PGT145 (n=2)	0.78 ± 0.017	1200 ± 300	0.92 ± 0.072	390 ± 70	0.74 ± 0.0027	3.2 ± 0.76	620 ± 180
PGT145 Fab (n=2)	0.60 ± 0.019	$1.8 \cdot 10^5$ $\pm 1.8 \cdot 10^5$	0.60 ± 0.023	$1.8 \cdot 10^5$ $\pm 1.8 \cdot 10^5$	0.59 ± 0.024	7.2 ± 2.8	430 ± 140
PGT151 (n=2)	1.8 ± 0.010	810 ± 35	2.0 ± 0.0090	490 ± 13	2.0 ± 0.010	95 ± 11	240 ± 140
PGT151 Fab (n=2)	2.0 ± 0.094	570 ± 28	2.0 ± 0.094	570 ± 28	1.9 ± 0.034	0.80 ± 0.067	1400 ± 80

[a] All tabulated values are the means ± s.e.m. of n independent experiments. The ligand is the BG505 SOSIP.664 trimer except in rows marked *protomer*.

[b] Stochiometry is calculated according to the formula $S_m = ((M_L)/(M_A)) \cdot ((R_{max})/(R_L))$, where M_L is the molar mass of the ligand, M_A the molar mass of the analyte, R_{max}, the modeled maximum binding signal for the analyte, and R_L the signal corresponding to the ligand capture. Since capture is repeated for every cycle with new ligand, local R_{max} fits are superior to global. The S_m values are based on the local R_{max} values with the highest T values (T_{max}), i.e. the most significant ones, are given.

[c] The S_m values based on the local R_{max} values for the highest concentrations of analyte (C_{max}), are given. Sometimes those R_{max} values coincide with the most significant ones. Agreement between the results obtained by the two local methods validates the S_m estimations.

[d] For comparison S_m values based on the globally fitted R_{max} values are given. These fits are moderately to markedly unsatisfactory, as indicated by the χ^2 values. The χ^2 values for local fits of IgG binding are given in Table S2. The significance (T values) of the globally determined R_{max} values are also given. How poor the global R_{max} fits are will depend on the random deviation in R_L from the ideal value of 500 RU among the cycles of each experiment. Therefore the agreement between the differently determined S_m values when the global fits are good validates the local methods.

Table S5. Bivalent modeling of IgG binding to BG505 SOSIP.664 trimer and gp120-gp41$_{ECTO}$ protomer

NAb (IgG)	k_{on1} [b] (1/Ms)	k_{off1} [b] (1/s)	k_{on2} [b] (1/RUs)	k_{off2} [b] (1/s)	K_{d1} [b] (nM)	K_{d2} [b] (RU)
VRC01 (n=2) [a]	$7.4 \cdot 10^3$ ± $1.1 \cdot 10^3$	$4.7 \cdot 10^{-6}$ ± $1.7 \cdot 10^{-6}$	$1.9 \cdot 10^{-3}$ ± $8.5 \cdot 10^{-4}$	$5.6 \cdot 10^{-2}$ ± $2.0 \cdot 10^{-2}$	0.72 ± 0.36	34 ± 7.3
VRC01 (n=2) *protomer*	$1.4 \cdot 10^4$ ± $3.5 \cdot 10^3$	$7.6 \cdot 10^{-7}$ ± $7.2 \cdot 10^{-8}$	$9.0 \cdot 10^{-4}$ ± $6.5 \cdot 10^{-4}$	$2.2 \cdot 10^{-2}$ ± $1.4 \cdot 10^{-2}$	0.061 ± 0.021	26 ± 3.3
PGV04 (n=3)	$3.9 \cdot 10^3$ ± 58	$< 10^{-5}$	$1.1 \cdot 10^{-3}$ ± $7.7 \cdot 10^{-4}$	$3.0 \cdot 10^{-2}$ ± $1.5 \cdot 10^{-2}$	<10	38 ± 10
PGT121 (n=3)	$1.2 \cdot 10^4$ ± $2.2 \cdot 10^3$	$8.5 \cdot 10^{-6}$ ± $1.1 \cdot 10^{-6}$	$3.3 \cdot 10^{-3}$ ± $2.7 \cdot 10^{-3}$	0.28 ± 0.26	0.76 ± 0.16	55 ± 23
PGT122 (n=2)	$4.6 \cdot 10^3$ ± $1.0 \cdot 10^2$	$3.0 \cdot 10^{-6}$ ± $2.2 \cdot 10^{-7}$	$2.4 \cdot 10^{-4}$ ± $3.1 \cdot 10^{-5}$	$5.6 \cdot 10^{-3}$ ± $2.1 \cdot 10^{-3}$	0.50 ± 0.063	23 ± 5.8
PGT122 (n=2) *protomer*	$4.9 \cdot 10^3$ ± $5.5 \cdot 10^2$	$3.0 \cdot 10^{-3}$ ± $2.2 \cdot 10^{-4}$	$6.9 \cdot 10^{-4}$ ± $6.2 \cdot 10^{-4}$	$1.4 \cdot 10^{-3}$ ± $1.2 \cdot 10^{-3}$	630 ± 120	2.4 ± 0.41
PGT123 (n=2)	$8.5 \cdot 10^3$ ± $3.3 \cdot 10^2$	$9.5 \cdot 10^{-6}$ ± $2.1 \cdot 10^{-6}$	$1.0 \cdot 10^{-3}$ ± $3.6 \cdot 10^{-4}$	$2.9 \cdot 10^{-2}$ ± $1.2 \cdot 10^{-2}$	1.11 ± 0.21	28 ± 2.2
PG9 (n=2)	$9.5 \cdot 10^3$ ± $2.9 \cdot 10^2$	$7.1 \cdot 10^{-4}$ ± $6.3 \cdot 10^{-5}$	$6.3 \cdot 10^{-3}$ ± $2.3 \cdot 10^{-3}$	$7.2 \cdot 10^{-3}$ ± $2.8 \cdot 10^{-3}$	75 ± 8.9	1.1 ± 0.019
PG16 (n=2)	$1.4 \cdot 10^4$ ± $6.0 \cdot 10^2$	$2.7 \cdot 10^{-3}$ ± $1.2 \cdot 10^{-4}$	$4.0 \cdot 10^{-3}$ ± $2.0 \cdot 10^{-4}$	$4.4 \cdot 10^{-2}$ ± $3.2 \cdot 10^{-3}$	190 ± 17	11 ± 0.25
PGT145 (n=2)	$2.4 \cdot 10^5$ ± $5.0 \cdot 10^3$	$6.9 \cdot 10^{-4}$ ± $2.1 \cdot 10^{-4}$	1.7 ± 1.7	0.11 ± 0.11	2.9 ± 0.94	$9.6 \cdot 10^{-2}$ ± $3.1 \cdot 10^{-2}$
PGT151 (n=2)	$5.5 \cdot 10^4$ ± $8.5 \cdot 10^2$	$3.5 \cdot 10^{-4}$ ± $2.0 \cdot 10^{-5}$	$1.4 \cdot 10^{-2}$ ± $2.8 \cdot 10^{-3}$	$2.6 \cdot 10^{-2}$ ± $4.0 \cdot 10^{-3}$	6.3 ± 0.47	2.0 ± 0.098

[a] All tabulated values are the means ± s.e.m. of n independent experiments. The ligand is the trimer, except in rows marked *protomer*.

[b] The kinetic constants were obtained by applying the bivalent model to IgG binding. In some cases fitting the off-rate constant for the initial interaction, k_{off1}, yielded T>10 only when the dissociation phase was extended to 20 min. Only those experiments were included in which significant values for k_{off1} were obtained. The on-rate constant for the initial interaction, k_{on1}, was always significant, and the kinetic constants for the second component nearly always so but that was not an inclusion criterion: their T values are shown in Table S2.

Table S6. Neutralization of BG505.T332N by Fabs

Fab	IC_{50} (nM) [b]	Fab/IgG IC_{50} [b]
PGV04 (n=2) [a]	3.5 (2.6-4.6)	6.0
PGT121 (n=2)	1.1 (0.79-1.4)	11
PGT123 (n=2)	2.1 (1.4-3.1)	12
PGT145 (n=2)	2.7 (1.8-4.0)	4.6

[a] The values in the table are derived from global non-linear regression fits to data from n replicate titrations for the Fabs; n values for IgG are in Table 1 in the main article.

[b] The Fab concentrations yielding a 2-fold reduction in relative infectivity were fitted with a conventional sigmoid function constrained to the respective relative top and bottom infectivity values of 1 and 0. The 95% confidence intervals for the IC_{50} values are converted from the modeled logarithmic values. The ratios of the IC_{50} values for Fab and IgG in nM were calculated.
