# Supplementary material for: Differential binding of neutralizing and non-neutralizing antibodies to native-like soluble HIV-1 Env trimers, uncleaved Env proteins, and monomeric subunits
Source: Retrovirology. 2014 May 29;11:41. doi: 10.1186/1742-4690-11-41 (PMC4067080; doi:10.1186/1742-4690-11-41)
Supplement: Additional file 4 — Supplementary results and commentary. [file 1742-4690-11-41-S4.pdf]

Differential binding of neutralizing and non-neutralizing antibodies to native-like soluble HIV-1 Env trimers, uncleaved Env proteins, and monomeric subunits

Anila Yasmeen[1], Rajesh Ringe[1], Ronald Derking[2],

Albert Cupo[1], Jean-Philippe Julien[3],

Dennis R. Burton[4, 5], Andrew B. Ward[3], Ian A. Wilson[3,6],

Rogier W. Sanders[1,2], John P. Moore[1], and P.J. Klasse[1 *]

[1] Department of Microbiology and Immunology, Weill Cornell Medical College, Cornell University, New York, USA

[2] Department of Medical Microbiology, Academic Medical Center, Amsterdam, The Netherlands

[3] Department of Integrative Structural and Computational Biology, International AIDS Vaccine Initiative Neutralizing Antibody Center and Center for HIV/AIDS Vaccine Immunology & Immunogen Discovery, The Scripps Research Institute, La Jolla, USA

[4] Department of Immunology and Microbial Science, International AIDS Vaccine Initiative Neutralizing Antibody Center, The Scripps Research Institute, La Jolla, CA, USA

[5] The Ragon Institute of Massachusetts General Hospital, Massachusetts Institute of Technology and Harvard University, Boston, MA, USA

[6] Skaggs Institute for Chemical Biology, The Scripps Research Institute, La Jolla, CA, USA

*pek2003@med.cornell.edu

Supplementary information

Comparisons of Env immobilization levels with spike densities on virions. We wanted to identify what determines antibody binding to native-like Env trimers. Since covalent coupling of trimers to dextran on CM5 chips perturbs the integrity of NAb epitopes and expose non-NAb epitopes (data not shown), we avoided such methods. Instead, every cycle started with capture of pristine Env protein. This approach, unavoidably, caused some variation in R_L (SD ~10%, Table S1).

R_L values of 500 RU for trimer and protomer and of 430 RU for gp120 were used so that the amount of gp120 subunits (mol) immobilized was kept approximately constant for the immobilizations of the different forms of Env (Table S1). An R_L value of 500 RU corresponds to 0.5 ng/mm^2 in the SPR detection area, equivalent to $1.2 \cdot 10^{-21}$ $mol/\mu m^2$, which translates into 700 trimer spikes per μm^2. The situation is simpler in this regard with immobilization to Ni^{2+}-NTA than to capture molecules linked to dextran on CM5 chips, because the dextran is flexible and fills out a small volume rather than presenting the trimer on a flat surface. For comparison, however, an HIV-1 virion has a diameter of 0.11 μm and hence an approximate surface area of 0.038 μm^2. The number of trimers on infectious virions, let alone of native-like or functional ones, is unknown, but a distribution of 4-35, with an average of 14, on the surface of HIV-1 MN virions was observed by cryo-EM (1). MN, however, is a T-cell-line-adapted strain of HIV-1, and therefore is likely to have fewer trimers per virion than most primary isolates ((2) and references therein). Furthermore, the virions with the fewest spikes may be non-infectious, and the observed trimers appeared in clusters over the virion surface. Because of these combined factors, a trimer density relevant to neutralization of primary isolates is probably in the upper range described or above it. The top of the range corresponds to 920 spikes per μm^2, i.e. 30% above the density we chose for standard SPR analyses. In experiments designed to address the conditions of bivalent binding, we also varied the R_L values of trimer captured onto Ni^{2+}-NTA.

It is noteworthy, however, that for some NAbs all forms of Env on the virion surface are relevant to potential bivalent binding; e.g., VRC01 might strengthen its

binding to the virion by cross-linking a functional and a non-functional spike. For other NAbs mainly the native forms are relevant (main article, Figure 1 and 2); e.g., PGT145 would probably only be able to cross-link native spikes and would therefore bind with lower avidity than VRC01, all other things being equal.

Comparisons of monovalent and bivalent binding to Env by SPR. The binding of IgG was fitted both with a simple Langmuir model and a more complex bivalent one. The Langmuir model describes the simplest bimolecular association, yielding k_{on} [1/Ms], k_{off} [1/s], and their ratio, K_d [M]. The bivalent model postulates a necessary initial one-point interaction (like that modeled by the Langmuir model) and an optional subsequent second-point interaction, i.e. the ligation of the antigen by the second paratope. The modeling adjudicates the extent of the second process. The initial interaction is described by the rate constants k_{on1} [1/Ms], k_{off1} [1/s], and their ratio, K_{d1} [M]. These parameters have the conventional dimensions for rate and equilibrium constants of a bimolecular associative reaction. The second component in the model consists of k_{on2} [1/RUs] and k_{off2} [1/s], yielding K_{d2} [RU]. These constants describe the strengthening of binding through bivalency. Since the bivalent interaction starts with an IgG molecule that already has bound to the antigen and given a resonance signal, the concentrations in this component are measured in RU. The bivalent model fitted the binding of IgG, both to trimer and, where studied, protomer better than the Langmuir model did (Table S2). Allowing for bivalency reduced the χ^2 value 2- to 12-fold.

Another way of assessing what difference the addition of the bivalent component makes is to compare the respective dissociation constants derived from the two models, K_d from Langmuir and K_{d1} from the bivalent model. The K_d of the Langmuir model describes the average binding of a molecule as if it were monovalent, whereas the K_{d1} of the bivalent model aims to dissect the affinity of a single binding site on the analyte for the corresponding site on the ligand. Thus the ratio K_{d1}/K_d becomes a measure of the apparent difference in functional and intrinsic affinity, or of what difference the inclusion of the bivalent component makes. This ratio ranged from 5.6 to $1.3 \cdot 10^4$ (Table S2). Before it can be assumed that the upper range of this wide interval accurately describes the functional affinity difference, the significance of the modeled parameters must also be

evaluated. The off-rate constant for the monovalent interaction in the bivalent modeling, k_{off1}, was often very low and therefore sometimes of borderline significance. By extending the dissociation phase, however, we could usually obtain significant values (T>10, T=mean/SD); and only experiments with significant values of k_{off1} are included in Table S2. The diagnostic for the need for bivalent modeling is that the off-rate constant for the Langmuir modeling, k_{off}, was still often very insignificant: T was < 10 (0.060-7.9) in six of the eleven cases in Table S2.

In addition, the T values for the two kinetic parameters of the second component were > 10 for the NAbs, or just below for PGT151, indicating that the modeling identifies significant bivalency, which validates the model. Possibly the bivalent binding of PGT151 is disfavored by the location of the epitope, which includes residues in one gp120 subunit and two gp41 moieties (3, 4).

In the few cases when both χ^2 and T values of the Langmuir modeling pass muster, the K_{d1}/K_d ratio would provide a means for assessing the avidity effect, i.e. of comparing the functional affinity of the IgG with the intrinsic affinity of the paratopes. Langmuir modeling of PGT122 against protomer and PG9, PGT145, and PGT151 against trimer gave χ^2 from 0.79 to 3.6 and T values for k_{off} from 37 to 100. The K_{d1}/K_d ratios from 5.6 to 74, calculated for these NAbs, would therefore have some legitimacy as avidity measurements, but of course only under the specific conditions of Env capture used in these experiments (Table S2).

The components of the bivalent model for all NAbs included in Table S2 are shown in Figure S3. Note that the bivalency described by the modeling is uniformly less prominent at the highest concentration than at lower ones. This is an example of the prozone effect; at the highest degrees of occupancy, approaching saturation, the second paratope of the IgG can less often find a free epitope to bind to.

We further explored the conditions for bivalent binding of VRC01 by varying the density of the SOSIP.664 trimer captured onto Ni^{2+}-NTA on the chip. Whereas a

reduction of R_L from 500 to 100 RU did not measurably reduce the bivalent component, an increase to 800 RU gave a near-complete bivalent interaction (Figure S4). We interpret this as meaning that at lower densities of trimer on the chip, clustering that does not linearly depend on ligand density allows some suboptimal bivalent interactions, but a modest increase (less than 2-fold) gives sufficiently small distances between trimers for nearly all IgG molecules to be able to cross-link. Since bivalent binding by NAbs to HIV-1 virions also appears to be partial and suboptimal (5), these results suggest that the SPR conditions (with a standard R_L of 500 RU) are relevant to neutralization.

A more direct way of measuring the avidity is to compare IgG and Fab binding. This also helps assessing how realistic the bivalent modeling is. For many Fab-IgG pairs the intrinsic affinity can be expected be similar, but exceptions caused by indirect effects on the paratope of distant parts of the IgG and by the accessibility to the epitope are noteworthy (6, 7).

The Langmuir modeling of the binding of VRC01 and PGT122 Fabs to both trimer and protomer and of PGV04, PGT123, PGT145, and PGT151 Fabs to trimer is compared with the corresponding monovalent components of IgG binding in Table S3. In addition, the predictably monovalent interaction of trimer as analyte with immobilized PGT145 IgG is compared there with the converse analyte-ligand format. Langmuir-modeled binding to the trimer by 2G12, which is functionally monovalent because of a domain-swap arrangement of its Fab arms, is also included in Table S3, but without comparison to other binding. Because the Langmuir-modeled value for k_{off} sometimes failed to reach significance in spite of prolonged dissociation phases (20 min), some estimates and comparisons could not be made. But it can be noted that none of the sets of Bivalent/Langmuir ratios of modeled or calculated constants, i.e. k_{on1}/k_{on}, k_{off1}/k_{off}, or K_{d1}/K_d deviated significantly from the ideal value of 1 (medians were, respectively: 0.87, 0.70, and 0.62, $p > 0.12$, Wilcoxon signed rank test). The monovalent interactions are shown in Figure S5 (except that of 2G12, which is included with the other IgGs in Figure 4). Note that PGT122 Fab, like the IgG version, binds markedly more weakly to the protomer than to the trimer.

In summary, monovalent and bivalent Ab binding can be kinetically analyzed, and both modes occur when IgG binds to captured trimeric or monomeric forms of Env at densities similar to what has been detected on the surface of virions. Bivalent strengthening of binding, or the avidity effect, can be quantified either by the modeled bivalency parameters or by comparing the modeled affinity of monovalent binding, i.e. intrinsic affinity, with the functional affinity of the whole IgG (Table S2). Bivalency will potentiate neutralization, and NAbs that can bind to both native-like and defective forms of Env (see Figures 1 and 2), may more easily find a nearest-neighbor for second-point attachment than would those NAbs that exclusively recognize functional trimers. The angle of approach to the trimer by a NAb is likely to affect the propensity for bivalent binding. The methods described here (Figure S4) also allow screening for rare NAbs capable of intra-trimeric bivalent binding, if any exist. That interaction would give a density-independent prominence of bivalent binding. And intra-trimeric bivalent binding, which should be confirmed by EM, would be advantageous by enhancing neutralization regardless of spike density on the virions.

Stoichiometric estimates of IgG binding to Env. The S_m value is the dimensionless stoichiometric estimate. Whether obtained by the Langmuir or the bivalent model, it gives the number of paratopes bound per Env molecule (trimer or protomer).

The inherent variation in R_L, even as low as in Table S1, often but not always precludes a precise global fitting of R_{max} for S_m determinations. (Global fitting of R_{max} is still appropriate for determining k_t reliably to exclude mass-transfer limitation as described in Methods.) When the S_m values are calculated from the local R_{max} values, the question arises whether the R_{max} obtained with the highest concentration of analyte or the most significant R_{max} value (highest T value) should be used. Although these R_{max} values tend to coincide, we observed some examples of when they did not, but then the most significant R_{max} was obtained for another high analyte concentration; typically T for R_{max} rises with analyte concentration. (T = the mean of a parameter value divided by the s.e.m. and indicates the significance of that value, as opposed to the overall fidelity of the

fit, assessed by χ^2.) We thus compared the S_m values obtained by the two local R_{max} fitting methods and the global one. As shown in Table S4, the agreement among the three methods was generally good, particularly when the global fitting gave a low χ^2. The method that gave the smallest variation in S_m among replicate experiments was the one based on the local fitting and the most significant R_{max} values. For later correlation analyses we used those values.

Kinetic modeling of the binding of IgGs with different stoichiometries. The results of the kinetic modeling for IgG versions of potentially bivalently binding NAbs are given in Table S5. The results for the CD4bs-directed NAbs VRC01 and PGV04, as well as the NAbs PGT121, PGT122, PGT123, and PGT151, are discussed in the main article. Three bNAbs directed to broadly similar, quaternary structure-dependent V1V2 glycan epitopes, PG9, PG16, and PGT145, displayed distinct kinetics (Figure 4, Table S5). PG9 had the more common pattern of moderate k_{on1} and low k_{off1}, yielding a K_{d1} value of 75 nM, which is 7-fold higher than the one derived for the corresponding Fab by ITC (8). This discrepancy may partly be explained by biochemical differences between the IgG and Fab preparations. Only the Fab was co-expressed with the tyrosine-sulfating enzyme TPST-1 and purified to homogeneity for the highest degree of sulfation (9). The PG16 profile was distinguished mainly by a higher k_{off1} and hence a higher K_{d1} value of 190 nM. Yet another pattern was seen with PGT145, which combined the highest k_{on1} among these three bNAbs with a k_{off1} similar to that for PG9, yielding the lowest K_{d1} value (2.9 nM).

The k_{on} value for PGT145 Fab ($2.1 \cdot 10^5$ (1/Ms)) agreed well with the k_{on1} value for the IgG ($2.4 \cdot 10^5$ (1/Ms)), whereas the k_{on} value for the trimer as analyte was ~ 2-fold lower (1.0 and $1.2 \cdot 10^5$ (1/Ms), Tables S3 and S5), possibly reflecting a difference in diffusion. The k_{off} of the Langmuir model can be compared with k_{off1} for IgG, and these values also agreed approximately, $4.2 \cdot 10^{-4}$ (1/s) for Fab, $6.2 \cdot 10^{-4}$ (1/s) for trimer as analyte, and $6.9 \cdot 10^{-4}$ (1/s) for IgG as analyte. Hence, the equilibrium-constant value for Fab (K_d = 2.0 nM) and for IgG binding monovalently to immobilized trimer (K_{d1} = 2.9 nM) were similar, but were 2- to 3-fold higher for trimer in solution when binding to immobilized IgG. As for PG9, the difference in sulfation between Fab and IgG (9) would

be expected to yield a higher affinity of the Fab, but such a difference was only detected for Fab compared with immobilized IgG, not compared with IgG as analyte.

In summary, the quaternary-structure-specific NAbs had distinct kinetic profiles and affinities, as could be expected from their different fine specificities and neutralizing capacity (9), but the stoichiometries were all compatible with a single paratope per trimer.

Caveats about SPR-ITC affinity comparisons. Several differences between the SPR and ITC methods might explain discrepant results. Affinity measurements by SPR (when based on kinetic modeling) are calculated as the k_{off}/k_{on} ratio, each constant having its own uncertainty. Thus, k_{off} may be so low as to be insignificant and high k_{on} values can be influenced by mass-transport limitation (which we took care to rule out, see Methods). SPR analyses require that either one of two molecules studied be immobilized. In most of the approaches in the current work, the tagged antigen (different forms of the Env protein) was immobilized by capture. In ITC both antigen and antibody are in solution. It cannot be excluded that there are subtle conformational differences between the captured and solution-phase forms of Env or that this would affect affinity measurements. Furthermore, the entropy changes would not be the same when one ligand binds to an already immobilized molecule as when two molecules in solution bind to each other.

BG505.T332N pseudovirus neutralization by Fabs. BG505.T332N pseudovirus was incubated with titrated Fabs, and residual infectivity was measured on Tzm-bl cells as previously described (12). The IC_{50}, calculated by fitting a sigmoid function with variable slope to the data, and Fab/IgG IC_{50} ratios are given in Table S6. The Fabs were 5- to 10-fold less potent than the IgGs, which suggests that bivalent binding to trimers by IgG occurs on the pseudovirion surface. Although the leveling off towards a lower plateau was less marked than for IgG, the extrapolated PFs tended to be similar for IgG and Fabs (data not shown). That bivalency has more pronounced effects on potency than efficacy might be explained by the well-established prozone effect that converts IgG binding to the monovalent mode at the highest occupancies (13). An exception to IgG-Fab similarity

in PF was PGT145. The extrapolated PF for the Fab was lower than for IgG; PGT145 also yielded the lowest Fab/IgG IC_{50} ratio. The explanation might be the different degrees of sulfation of Fab and IgG (9).

Statistical analyses. The deviations of the monovalent kinetic and dissociation constants obtained by the bivalent modeling for IgG from the corresponding ones obtained by the Langmuir modeling for Fabs were analyzed by Wilcoxon signed rank test.

The fitted parameters of the bivalent model were analyzed by first ranking all values in descending order. All ranked values, as expected, passed the Kolmogorov-Smirnoff, d'Agostino-Pearson, and Shapiro-Wilk normality tests. After ranking we used Pearson correlations with two-tailed tests for significance. After initial analysis, we also correlated the ranked PF values with the sum of the ranked k_{off1} and the S_m values ranked in ascending order.

Supplementary references

1. **Zhu P, Liu J, Bess J, Jr., Chertova E, Lifson JD, Grise H, Ofek GA, Taylor KA, Roux KH.** 2006. Distribution and three-dimensional structure of AIDS virus envelope spikes. Nature **441:**847-852.
2. **Klasse PJ, Moore JP.** 1996. Quantitative model of antibody- and soluble CD4-mediated neutralization of primary isolates and T-cell line-adapted strains of human immunodeficiency virus type 1. J Virol **70:**3668-3677.
3. **Blattner C, Lee JH, Sliepen K, Derking R, Falkowska E, de la Pena AT, Cupo A, Julien JP, van Gils M, Lee PS, Peng W, Paulson JC, Poignard P, Burton DR, Moore JP, Sanders RW, Wilson IA, Ward AB.** 2014. Structural Delineation of a Quaternary, Cleavage-Dependent Epitope at the gp41-gp120 Interface on Intact HIV-1 Env Trimers. Immunity.
4. **Falkowska E, Le KM, Ramos A, Doores KJ, Lee JH, Blattner C, Ramirez A, Derking R, van Gils MJ, Liang CH, McBride R, von Bredow B, Shivatare SS, Wu CY, Chan-Hui PY, Liu Y, Feizi T, Zwick MB, Koff WC, Seaman MS, Swiderek K, Moore JP, Evans D, Paulson JC, Wong CH, Ward AB, Wilson IA, Sanders RW, Poignard P, Burton DR.** 2014. Broadly Neutralizing HIV Antibodies Define a Glycan-Dependent Epitope on the Prefusion Conformation of gp41 on Cleaved Envelope Trimers. Immunity.
5. **Klein JS, Bjorkman PJ.** 2010. Few and far between: how HIV may be evading antibody avidity. PLoS Pathog **6:**e1000908.
6. **Labrijn AF, Poignard P, Raja A, Zwick MB, Delgado K, Franti M, Binley J, Vivona V, Grundner C, Huang CC, Venturi M, Petropoulos CJ, Wrin T, Dimitrov DS, Robinson J, Kwong PD, Wyatt RT, Sodroski J, Burton DR.** 2003. Access of antibody molecules to the conserved coreceptor binding site on glycoprotein gp120 is sterically restricted on primary human immunodeficiency virus type 1. J Virol **77:**10557-10565.
7. **Crespillo S, Casares S, Mateo PL, Conejero-Lara F.** 2014. Thermodynamic analysis of the binding of 2F5 (Fab and immunoglobulin G forms) to its gp41 epitope reveals a strong influence of the immunoglobulin Fc region on affinity. J Biol Chem **289:**594-599.
8. **Julien JP, Lee JH, Cupo A, Murin CD, Derking R, Hoffenberg S, Caulfield MJ, King CR, Marozsan AJ, Klasse PJ, Sanders RW, Moore JP, Wilson IA, Ward AB.** 2013. Asymmetric recognition of the HIV-1 trimer by broadly neutralizing antibody PG9. Proc Natl Acad Sci U S A **110:**4351-4356.
9. **Pejchal R, Walker LM, Stanfield RL, Phogat SK, Koff WC, Poignard P, Burton DR, Wilson IA.** 2010. Structure and function of broadly reactive antibody PG16 reveal an H3 subdomain that mediates potent neutralization of HIV-1. Proc Natl Acad Sci U S A **107:**11483-11488.
10. **Klasse PJ.** 2007. Modeling how many envelope glycoprotein trimers per virion participate in human immunodeficiency virus infectivity and its neutralization by antibody. Virology **369:**245-262.
11. **Magnus C, Regoes RR.** 2010. Estimating the stoichiometry of HIV neutralization. PLoS Comput Biol **6:**e1000713.
12. **Sanders RW, Derking R, Cupo A, Julien JP, Yasmeen A, de Val N, Kim HJ, Blattner C, de la Pena AT, Korzun J, Golabek M, de Los Reyes K, Ketas TJ,**

van Gils MJ, King CR, Wilson IA, Ward AB, Klasse PJ, Moore JP. 2013. A next-generation cleaved, soluble HIV-1 Env Trimer, BG505 SOSIP.664 gp140, expresses multiple epitopes for broadly neutralizing but not non-neutralizing antibodies. PLoS Pathog **9:**e1003618.

13. **Vos Q, Klasen EA, Haaijman JJ.** 1987. The effect of divalent and univalent binding on antibody titration curves in solid-phase ELISA. J Immunol Methods **103:**47-54.
